# Kinetics of DNA strand transfer between polymerase and proofreading exonuclease active sites regulates error correction during high-fidelity replication

**DOI:** 10.1016/j.jbc.2022.102744

**Published:** 2022-11-24

**Authors:** Tyler L. Dangerfield, Kenneth A. Johnson

**Affiliations:** Department of Molecular Biosciences, University of Texas, Austin, Texas, USA

**Keywords:** DNA polymerase, pre–steady-state kinetics, nucleic acid enzymology, exonuclease, proofreading, stopped flow, fluorescence, enzyme mechanism, exo, exonucleae, FAM, carboxyfluorescein, pol, polymerase, tC^o^, 1,3-diaza-2-oxophenoxazine, T4 PNK, T4 polynucleotide kinase

## Abstract

We show that T7 DNA polymerase (pol) and exonuclease (exo) domains contribute to selective error correction during DNA replication by regulating bidirectional strand transfer between the two active sites. To explore the kinetic basis for selective removal of mismatches, we used a fluorescent cytosine analog (1,3-diaza-2-oxophenoxazine) to monitor the kinetics of DNA transfer between the exo and pol sites. We globally fit stopped-flow fluorescence and base excision kinetic data and compared results obtained with ssDNA *versus* duplex DNA to resolve how DNA transfer governs exo specificity. We performed parallel studies using hydrolysis-resistant phosphorothioate oligonucleotides to monitor DNA transfer to the exo site without hydrolysis. ssDNA binds to the exo site at the diffusion limit (10^9^ M^−1^ s^−1^, K_d_ = 40 nM) followed by fast hydrolysis of the 3′-terminal nucleotide (>5000 s^−1^). Analysis using duplex DNA with a 3′-terminal mismatch or a buried mismatch exposed a unique intermediate state between pol and exo active sites and revealed that transfer *via* the intermediate to the exo site is stimulated by free nucleoside triphosphates. Transfer from the exo site back to the pol site after cleavage is fast and efficient. We propose a model to explain why buried mismatches are removed faster than single 3′-terminal mismatches and thereby provide an additional opportunity for error correction. Our data provide the first comprehensive model to explain how DNA transfer from pol to exo active sites and back again after base excision allow efficient selective mismatch removal during DNA replication to improve fidelity by more than 1000-fold.

DNA polymerase (pol) fidelity is finely tuned to support the survival of most organisms. For many viral enzymes such as HIV reverse transcriptase, a pol with only moderate fidelity (one error in 10^4^) strikes the right balance between replication that is accurate enough to propagate the virus but with a sufficient error rate to rapidly evade the host’s immune response. For high-fidelity enzymes, the net accuracy is a product of the intrinsic fidelity during nucleotide incorporation at the pol active site and the contribution of a 3′-5′ proofreading exonuclease (exo) that selectively removes mismatched nucleotides to further improve fidelity. For the well-characterized high-fidelity T7 DNA pol, the proofreading exo domain binds ssDNA and is 25 Å away from the pol active site. Therefore, a major challenge is to understand how the exo achieves specificity to remove mismatches when the DNA is no longer base-paired with the template strand when in the exo active site. The enzyme solves the problem by modulating exo hydrolysis by kinetic partitioning of the primer strand between forward polymerization *versus* transfer of the primer strand to the exo site. Following incorporation of a mismatch, the partitioning changes because of much slower forward polymerization and slightly faster transfer of the primer stand to the exo site (1, 2). Once in the exo active site, the 3′-terminal nucleotide is rapidly excised, and then the primer strand transfers back to the pol active site to allow continuing processive polymerization. Thus, the proofreading exo achieves specificity indirectly based on the events occurring at the pol active site that regulate the partitioning between polymerization to extend *versus* hydrolysis to remove the 3′-terminal base pair. Although this model was derived 3 decades ago and provides a satisfying resolution of the conundrum, little is known regarding the kinetics of primer strand transfer between the pol and exo active sites despite numerous studies (3, 4, 5, 6, 7).

We previously reported methods to label the T7 DNA pol with a fluorescent artificial amino acid that gives a fluorescence change upon nucleotide binding (8). We then used this variant to characterize the conformational dynamics that govern correct nucleotide incorporation (9) and compared that with mismatched nucleotide incorporation (10) to define the mechanistic basis for the extraordinary fidelity of this enzyme. We also previously characterized the DNA substrate specificity for the proofreading exo of T7 DNA pol using rapid-quench methods and showed that the rate of mismatch excision increased with the single-stranded nature of the DNA substrate; that is, ssDNA was hydrolyzed the fastest, whereas the 3′-terminal base in duplex DNA was removed at a rate that increased with the number of mismatched base pairs (11). Surprisingly, we found that a mismatch buried by a single correct base pair was removed much more efficiently than a single terminal mismatch, demonstrating that the enzyme affords multiple opportunities to remove a mismatch. We also presented a structural model for the proofreading complex for T7 DNA pol based on homology to a crystal structure of a Klenow fragment of *Escherichia coli* DNA pol I editing complex, which showed that three bases must melt for the primer strand to reach fully into the exo active site. Based on the observation that the stereochemistry of the exo hydrolysis reaction is the same as that for similar pols that have been characterized, we proposed a catalytic mechanism for exo hydrolysis (10, 11). This study, however, left unanswered questions about the detailed kinetics governing partitioning of the DNA between the two active sites. Because of hydrolysis of ssDNA at least 100-fold faster than removal of a 3′-terminal mismatch from duplex DNA, the rate of transfer of the DNA from the pol to the exo site appears to limit the rate of hydrolysis.

In this article, we expand on our previous studies on exo proofreading by T7 DNA pol through characterization of exo hydrolysis on three physiologically relevant substrates: ssDNA, DNA containing a single 3′ mismatch, and DNA containing a buried mismatch. To measure DNA binding and dissociation kinetics at the exo active site, we enzymatically synthesized hydrolysis-resistant DNA substrates containing phosphorothioate nucleotides—this is preferred to the alternative method of using exo-deficient mutants derived by removal of charged residues that bind Mg^2+^ and thereby perturb the electrostatics at the active site. We also introduce a DNA substrate containing the fluorescent cytosine analog 1,3-diaza-2-oxophenoxazine (tC^o^) that provides a large fluorescence signal upon transitioning from dsDNA in the pol active site to ssDNA in the exo active site. We provide new data to define the kinetics of the transfer of DNA between the pol and exo active sites.

We first show that excision of ssDNA in the exo site is very fast; so in subsequent experiments with dsDNA, the observed rates of excision are limited by, and thereby serve as a readout for the rates of transfer of the primer strand to the exo site. Fluorescence signals are used to monitor DNA binding (carboxyfluorescein [FAM] label anisotropy) and unwinding (tC^o^ signal), whereas rapid-quench methods define the kinetics of nucleotide excision. We used two protocols to explore DNA binding to the pol and exo sites: (1) mixing enzyme and DNA to initiate the reaction provides evidence to define kinetics of binding to pol and exo sites; (2) by preincubating enzyme and DNA without Mg^2+^, then initiating the reaction by adding Mg^2+^ gives data to define the equilibrium distribution between binding states and the kinetics of partitioning between the sites, as evidenced by fluorescence and rapid-quench kinetic data; (3) by adding nucleotides that extend the DNA only after removal of a mismatch, we obtain information to define the kinetics of transfer to the exo site and back to the pol site. In these experiments, we know that polymerization is very fast, and so the observed rates appear to be limited by the transfer between the active sites. Naturally, each of these experiments provides information on the multiple binding states and kinetics of transfer between the states, but no single experiment can be interpreted directly. Rather, it is only through global data fitting that the relationships between DNA binding and unwinding can be correlated with the kinetics of strand transfer and nucleotide excision to define a comprehensive model to define the molecular basis for selective mismatch removal by the proofreading exo.

## Results

### Use of the fluorescent cytosine analog (tC^o^) to measure DNA binding and melting of dsDNA

**Figure 1:**
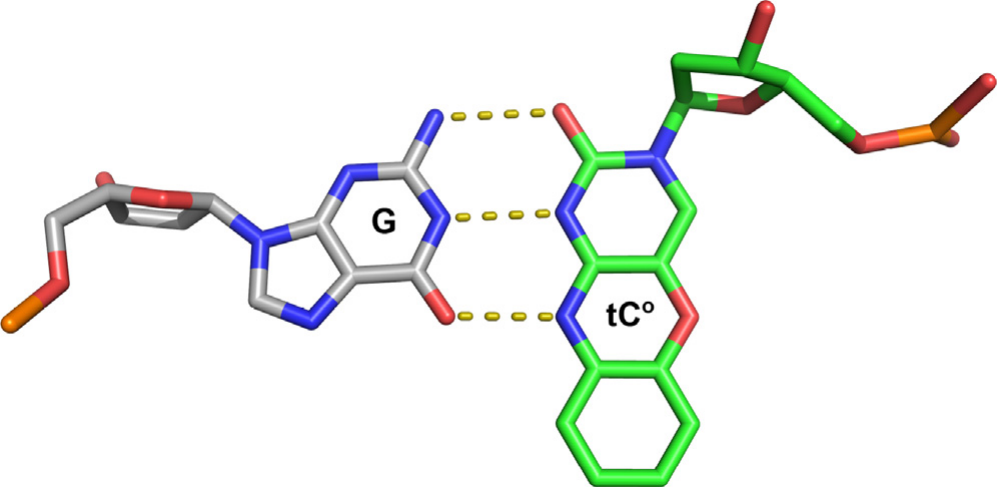
**Fluorescent cytosine analog, tC**^**o**^**, as a probe for DNA melting to monitor the transfer between exonuclease and polymerase active sites.** Base pairing between G (*gray*) and tC^o^ (*green*) is shown, with three hydrogen bonds as expected for a G:C base pair. The fluorescence of tC^o^ increases when the duplex DNA melts to form to ssDNA. tC^o^, 1,3-diaza-2-oxophenoxazine.

**Figure 2:**
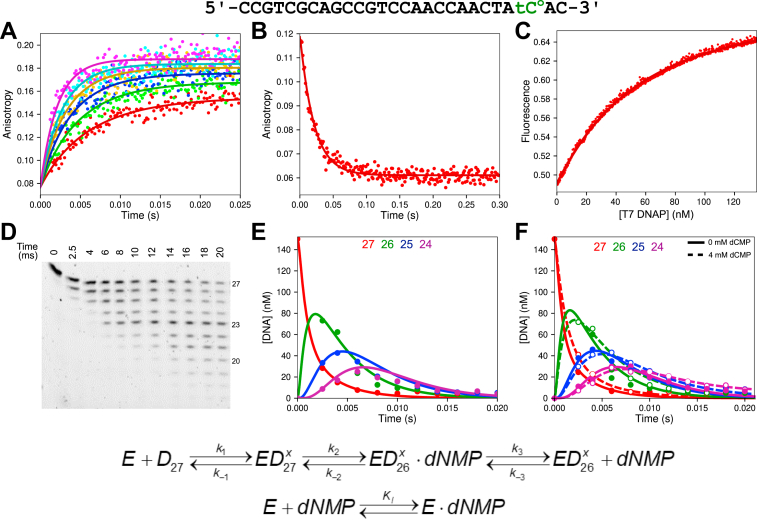
**Kinetics of ssDNA binding and excision.** DNA substrate: The 27 nt ssDNA substrate used in these experiments is shown at the *top* of this figure. Experiments with 6-FAM-labeled DNA contained a 5’ [6-FAM] label and a C rather than tC^o^. Experiments in (*A*) through (*C*) were performed using a hydrolysis-resistant phosphorothioate (S_p_ isomer) ssDNA (see the Experimental procedures section). Below, we describe approximate estimates of kinetic parameters derived from equation-based data fitting, but the *solid lines* through the data show the best global fits with KinTek Explorer using the model at the *bottom* of the figure and parameters listed in Table 2. *A*, stopped-flow ssDNA binding rate. A solution of 50 nM FAM-phosphorothioate ssDNA (FAM-27-PThio) and 12.5 mM Mg^2+^ was mixed with varying concentrations of T7 DNA polymerase (100–400 nM) to start the reaction, and the change in anisotropy as a function of time was monitored. This experiment provides an estimate of the ssDNA binding rate constant (*k*_*1*_) of ∼1000 μM^−1^ s^−1^, and a significant dissociation rate constant (*k*_*-1*_ ∼ 40 s^−1^), defined by the amplitude dependence of the reaction. *B*, stopped-flow ssDNA off-rate experiment. A solution of 50 nM FAM-27-PThio, 75 nM T7 DNA polymerase, and 12.5 mM Mg^2+^ was mixed with 1 μM unlabeled phosphorothioate ssDNA as a trap to start the reaction, and the anisotropy as a function of time is shown. The data best fit a single exponential function with an observed rate of 47.5 ± 1.5 s^−1^. *C*, stopped-flow ssDNA equilibrium titration. A solution of 25 nM tC^o^ phosphorothioate ssDNA (tC^o^-27-PThio) and 12.5 mM Mg^2+^ was titrated with T7 DNA polymerase over the course of 5 min with constant stirring using a micro stir bar. The fluorescence was corrected for the small dilution and an inverse inner filter effect as described in the Experimental procedures section. The data fit a hyperbola giving a *K*_*d*_ ∼60 nM. *D*, rapid-quench ssDNA excision kinetics—gel. A solution of 150 nM FAM ssDNA (FAM-27) and 12.5 mM Mg^2+^ was mixed with varying concentrations of T7 DNA polymerase (0.25–1.25 μM) and 0.1 mg/ml BSA to start the reaction. Samples were quenched with EDTA at various times, and products were resolved by denaturing PAGE (15% acrylamide). Lengths of ssDNA are given to the *right* of the gel. The gel from the experiment at 0.25 μM T7 DNA polymerase is shown as a representative dataset. *E*, rapid-quench ssDNA excision kinetics—concentration *versus* time. Reaction conditions are given in (*D*). Data corresponding to 27, 26, 25, and 24 nt ssDNA are shown in *red*, *green*, *blue*, and *magenta*, respectively. Other bands are omitted for clarity. The experiment at 1.25 μM T7 DNA polymerase is shown as a representative dataset. *F*, rapid-quench ssDNA excision, inhibition by dCMP. A solution of 1 μM T7 DNA polymerase, 0.1 mg/ml BSA, and 12.5 mM Mg^2+^ was mixed with 150 nM FAM ssDNA (FAM-27) and either 0 or 4 mM dCMP to start the reaction. Samples were quenched with EDTA, and products were resolved by PAGE as above. Data at 4 mM dCMP are shown as *open circles* with *dashed lines* for the fit by simulation, whereas data at 0 mM dCMP are shown with *filled circles* and *solid lines* for the fit by simulation. Scheme: Kinetic model for ssDNA binding and excision. The x superscript following the D indicates binding at the exo site, and the numbered subscript indicates the length of the DNA substrate. 6-FAM, 6-carboxyfluorescein; tC^o^, 1,3-diaza-2-oxophenoxazine; BSA, bovine serum albumin.

We previously described kinetics of exo hydrolysis on various DNA substrates using rapid-quench methods (11). These studies, while useful to determine relative observed rates of excision on different DNA substrates, do not provide sufficient information to determine rate constants for individual steps governing the exo proofreading reaction. In particular, to monitor transfer of the DNA between the pol and exo active sites, we prepared a DNA substrate containing the fluorescent cytosine analog tC^o^ at the n-2 position from the 3′ end of the primer strand (Figs. 1 and 2). The analog tC^o^ has some unique properties that make it an ideal fluorescent probe relative to other available fluorescent nucleoside analogs, such as 2-aminopurine—it has a quantum yield of 0.3 when in ssDNA oligonucleotides, which is reduced to 0.22 in dsDNA substrates, making tC^o^ the brightest DNA base analog so far reported, and it provides a significant change in signal to monitor DNA unwinding (12). Furthermore, studies have shown that tC^o^ base pairs almost exclusively with guanine, causes minimal perturbations to the native structure of DNA (12), and CD spectra showed that all tC^o^ containing oligonucleotide duplexes form the characteristic B-form DNA. Other fluorescent DNA base analogs share a common feature in that the emission intensity is highly sensitive to microenvironment, whereas tC^o^ is relatively insensitive to its local chemical environment (12) but is quenched by base-stacking interactions. Furthermore, Klenow fragment of *E. coli* DNA pol I and human DNA pol α have been shown to incorporate the tC^o^ analog with high efficiency (13, 14).

We chose the n-2 position in the primer strand to place the tC^o^ label based on our homology model for the exo proofreading complex that we described previously (11). This model clearly showed that three bases must melt for the primer strand to partition completely into the exo active site, so the n-2 position where we incorporated tC^o^ should undergo a double-strand to single-strand transition upon complete partitioning into the exo active site, giving rise to a fluorescence change. In addition, by placing the fluorescent base analog as the third base from the primer terminus, we reasoned that the analog is less likely to cause significant perturbations affecting DNA binding in the exo site and partitioning of the primer strand. We also carefully selected the surrounding sequences using a table from a previous study showing that tC^o^ surrounded by an A on either side has negligible effect on the melting temperature of the duplex (12). As a control experiment to test whether the tC^o^ label affected normal polymerization kinetics, we performed a rapid-quench pre–steady-state burst experiment (Fig. S1), where the next correct nucleotide was added to the primer strand. This experiment demonstrated that T7 DNA pol is still able to rapidly add bases to a primer containing tC^o^ at rates exceeding 300 nt s^−1^ and with full amplitude, as defined in studies using normal nucleotides (9).

### Hydrolysis-resistant phosphorothioate oligonucleotides to study DNA binding

In our previous article on exo proofreading (11), we determined the stereospecificity of the T7 DNA exo using phosphorothioate DNA and showed that the S_p_ isomer was resistant to hydrolysis. This is consistent with the stereochemistry of the reactions for T4 DNA pol (15) and Klenow fragment of *E. coli* DNA pol I (16, 17). Using this information, we developed methods to enzymatically degrade the R_p_ isomer in a racemic mixture of both diastereomers (see the Experimental procedures section), followed by purification of the resulting hydrolysis-resistant oligonucleotide by ion exchange chromatography. The presence of full-length oligonucleotide was then confirmed by MALDI-MS. Although previous studies on other enzymes (16, 17) have shown that the S_p_ phosphorothioate is not a perfect substrate for exo domains as it interferes with metal binding at the active site, we opted to use the phosphorothioate approach to measure DNA binding rather than the alternative of performing studies in the absence of Mg^2+^ or with active-site mutants. Preliminary studies with the exo-T7 DNA pol variant showed large differences in DNA binding and dissociation kinetics between experiments with and without Mg^2+^ (not shown), whereas the difference between normal and phosphorothioate DNA in the presence of Mg^2+^ was minimal.

### Kinetics of ssDNA binding

Since ssDNA is the preferred substrate for the proofreading exo (2), we began our detailed kinetic studies on exo proofreading using this substrate. The physiologically relevant substrate is duplex DNA with a melted ssDNA 3′-strand projecting into the exo site; therefore, ssDNA provides a useful model for understanding the reactions of DNA in the exo site. Before investigating the kinetics of excision, we used hydrolysis-resistant phosphorothioate (S_p_ isomer) single-stranded oligonucleotides to measure binding kinetics without hydrolysis. We first measured the ssDNA binding rate by stopped-flow fluorescence using the anisotropy signal from a 6-FAM-labeled phosphorothioate ssDNA substrate (FAM-27-PThio in Table 1). In this experiment, a fixed concentration of FAM-labeled phosphorothioate ssDNA was mixed with varying concentrations of T7 DNA pol (with enzyme in excess) to start the reaction, and we then monitored the change in anisotropy as a function of time (Fig. 2*A*). The rapid timescale of the experiment indicates that the binding rate is extremely fast, with a second-order rate constant of ∼1000 μM^−1^ s^−1^. The observed amplitude dependence on enzyme concentration suggests that the equilibrium dissociation constant for ssDNA is comparable to the enzyme concentrations covered in the experiment. We then directly measured the ssDNA dissociate rate using the anisotropy signal from the FAM-labeled phosphorothioate ssDNA (Fig. 2*B*). A solution of T7 DNA pol and FAM-labeled phosphorothioate ssDNA was mixed with a large excess of unlabeled phosphorothioate ssDNA in the stopped-flow to start the reaction. The unlabeled phosphorothioate ssDNA acts as a trap for any enzyme that has dissociated from the T7 DNA pol–FAM-labeled ssDNA complex. The data best fit a single exponential function with an observed rate of 48 ± 1.5 s^−1^ (fit not shown). This fast off rate is consistent with the amplitude dependence observed in the ssDNA binding rate experiment. Simultaneous global fitting of binding and dissociation data resolves both constants (Table 2). Finally, to ensure all binding steps have been accounted for, an equilibrium titration experiment was performed using the tC^o^ phosphorothioate ssDNA. In this experiment, a solution of tC^o^ phosphorothioate ssDNA was incubated in a temperature-controlled cuvette, and T7 DNA pol was titrated over the course of 5 min with constant mixing from a micro stir bar. Since the signal for ssDNA binding using the tC^o^ phosphorothioate substrate was small when exciting tC^o^ directly at 365 nm, we opted to use the FRET signal between tryptophan residues in T7 DNA pol and the tC^o^ label by exciting the sample at 295 nm, which produced a large signal upon DNA binding. The titration data were corrected for the small dilution and the fluorescence artifact from titration of the enzyme (FRET donor) as described in the Experimental procedures section to give the corrected data shown in Figure 2*C*. The titration curve best fits a hyperbola with a *K*_*d*_ for ssDNA binding of approximately 60 nM. All binding data were fit globally in KinTek Explorer (KinTek Corporation; kintekexplorer.com), and the resulting fits are shown as the *solid lines* through the data in Figure 2, *A*–*C* with rate constants given in Table 2 with *k*_*1*_ = 1100 ± 20 μM^−1^ s^−1^, *k*_*-1*_ = 44 ± 1.3 s^−1^, and *K*_*d*_ = 40 ± 1.4 nM.

**Table 1:** Oligonucleotides used in this study

Oligo name	Sequence 5’→3′	Extinction coefficient, 260 nm (M^−1^ cm^−1^)
FAM-27	[6-FAM]-CCGTCGCAGCCGTCCAACCAACTCAAC	266,660
FAM-27-PThio	[6-FAM]-CCGTCGCAGCCGTCCAACCAACTACA∗C	267,660
27	CCGTCGCAGCCGTCCAACCAACTACAC	246,700
27-PThio	CCGTCGCAGCCGTCCAACCAACTACA∗C	246,700
tC^o^-27	CCGTCGCAGCCGTCCAACCAACTA[tC^o^]AC	250,100
^32^P-tC^o^-27	^32^P-CCGTCGCAGCCGTCCAACCAACTA[tC^o^]AC	250,100
tC^o^-27-PThio	CCGTCGCAGCCGTCCAACCAACTA[tC^o^]A∗C	250,100
45-P/T	GGACGGCATTGGATCGATGTGTAGTTGGTTGGACGGCTGCGACGG	435,100
45-3′MM	GGACGGCATTGGATCGATCTGTAGTTGGTTGGACGGCTGCGACGG	431,600
45-3′MM-PThio	GGACGGCATTGGATCGATCTGTAGTTGGTTGGACGGCTGCGACG∗G	431,600
45-BMM	GGACGGCATTGGATCGATGAGTAGTTGGTTGGACGGCTGCGACGG	439,600
45-BMM-PThio	GGACGGCATTGGATCGATGAGTAGTTGGTTGGACGGCTGCGACG∗G	439,600

We use ∗ to designate the phosphorothioate linkage.

**Table 2:** Kinetic parameters for ssDNA^a^

Parameter	Best fit	95% Confidence interval
*k*_*1*_	1100 μM^−1^ s^−1^	(1070, 1130)
*k*_*-1*_	44.1 s^−1^	(42.8, 45.5)
*k*_*2*_	**5000 s^−1^**	Locked
*k*_*-2*_	1670 s^−1^	(1470, 1920)
*k*_*3*_	443 s^−1^	(415, 480)
*k*_*-3*_	0.0518 μM^−1^ s^−1^	(0.0374, 0.0648)
*k*_*3,2*_	546 s^−1^	(516, 584)
*k*_*3,3*_	631 s^−1^	(591, 675)
*K*_*I*_	5.65 mM	(4.52, 7.06)

Rates constants shown in bold font were locked at the displayed value during global data fitting.

a Rate constants derived in Figure 2 are summarized with confidence intervals derived by contour analysis. Because the contours are symmetrical, we can estimate the standard errors directly from the confidence intervals, for example, *k*_*1*_ = 1100 ± 30 μM^−1^ s^−1^. The rate constant *k*_*2*_ was locked at a lower limit of 5000 s^−1^. Rate constants *k*_*3*_, *k*_*3,2*_, and *k*_*3,3*_ are for the release of sequential dNMP products from the 3′-end of substrate FAM-27 shown in Table 1 (*i.e.*, dCMP, dAMP, and dAMP).

### Kinetics of ssDNA hydrolysis

Next, we determined the kinetics of ssDNA hydrolysis using rapid-quench methods. A solution of FAM-ssDNA was mixed with varying concentrations of T7 DNA pol (with the enzyme in excess and therefore determining the observed rate) to start the reaction. Samples were quenched with EDTA at various times, and products were resolved by denaturing PAGE. A representative gel is shown in Figure 2*D*. Preliminary inspection of the gel clearly reveals a fast rate of excision of ssDNA by T7 DNA pol, with eight bases removed in less than 20 ms. By performing the experiment at multiple enzyme concentrations, we obtained estimates of the rate constant for DNA binding in agreement with the measurements made using the phosphorothioate DNA. This analysis afforded resolution of the rate constants for DNA binding and chemistry (*k*_*1*_ and *k*_*2*_, respectively, in the scheme in Fig. 2).

A representative plot of concentration of product *versus* time at a single enzyme concentration is given in Figure 2*E*, showing products from 27 to 24 nt in length (shorter products were omitted for clarity). The data best fit a model with fast ssDNA binding (∼1000 μM^−1^ s^−1^) and an even faster rate of chemistry (>5000 s^−1^). In other words, the observed excision kinetics for the first hydrolyzed base at all enzyme concentrations tested was limited by the rate of ssDNA binding. Fitting the data also demonstrated that the first nucleotide was hydrolyzed faster than subsequent bases, leading to a model where ssDNA binds, the terminal base is rapidly hydrolyzed, and release of the dNMP product (*k*_*3*_ in the scheme in Fig. 2) limits the rate of subsequent excision reactions (∼500 s^−1^), but the cleavage rate varies depending on the identity of the terminal base (440–630 s^−1^). Rates of dNMP release extracted from the fitting for excision of the first three bases are given in Table 2.

In our previous work on correct nucleotide insertion for T7 DNA pol (9), we were able to measure rates of the pyrophosphorolysis reaction (reverse of nucleotide incorporation), which gave insights into the translocation step that was not observable when monitoring the reaction in the forward direction. Since the reverse reaction for exo hydrolysis is not observable even at long timescales (data not shown), we performed the exo hydrolysis experiment (forward reaction) in the presence of dCMP in an attempt to extract the second-order rate constant for dNMP binding (*k*_*-3*_ in the scheme in Fig. 2) and the reverse of chemistry (*k*_*-2*_ in the scheme in Fig. 2). The results are shown in Figure 2*F* (*dashed lines*). In this experiment, a solution of FAM-ssDNA was mixed with an excess of T7 DNA pol and either 0 or 4 mM dCMP to start the reaction in the rapid-quench flow instrument, and samples collected by quenching at various times were processed as described previously. The data indicate that dCMP binding is relatively slow (*k*_*-3*_ = 0.052 μM^−1^ s^−1^), but the data afford an estimate for the equilibrium constant for the chemistry step. Since the rate constant for chemistry is too fast to measure, we locked this rate constant at a lower limit of 5000 s^−1^ and fit the data to estimate the rate constant for the reverse of chemistry (*k*_*-2*_ = 1670 s^−1^) resulting in an equilibrium constant for the chemistry step, *K*_*2*_ = 3.4. Fitting the data also required including a step where dCMP binds to the free enzyme, acting as a weak competitive inhibitor for ssDNA binding (*K*_*i*_ in the scheme in Fig. 2). To estimate the equilibrium constant, we locked the rate of dNMP binding to the free enzyme at 100 μM^−1^ s^−1^ and allowed the dNMP off rate to float to extract a *K*_*i*_ of 5.6 mM.

**Figure 3:**
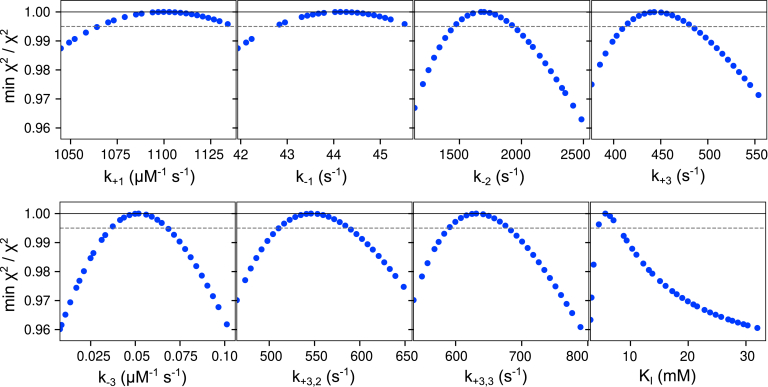
**Confidence contours for ssDNA binding and excision kinetics.** Confidence contours from the FitSpace calculation in KinTek Explorer are shown for the global fit of all ssDNA experiments in Figure 2. *k*_*2*_ was locked at 5000 s^−1^ in the fitting. The *dashed line* gives the 99.5% threshold, corresponding to the 95% confidence interval based on the number of data points and parameters in the fitting, as calculated by KinTek Explorer. Best-fit parameters and confidence intervals are given in Table 2.

All experiments for ssDNA were fit globally using the model in Figure 2, and we then tested whether each parameter was well constrained by the data based on confidence contour analysis with the FitSpace feature of KinTek Explorer (18) as shown in Figure 3. This statistical analysis is the most rigorous test to determine whether parameters in the model are well constrained by the data and circumvents many of the pitfalls of error analysis from nonlinear regression when parameters are linked or are not well constrained (19). The contours are obtained by measuring the dependence of the χ^2^ on each parameter while allowing all remaining parameters to float in finding the optimal fit. The parabolic confidence contour curves outline the space over which parameters can vary and indicate that each parameter was well constrained by the data—the *dashed line* each panel gives the threshold corresponding to the 95% confidence interval for each parameter, calculated based on the number of parameters and number of data points in the fitting (19).

### Kinetics of 3′ mismatch hydrolysis

**Figure 4:**
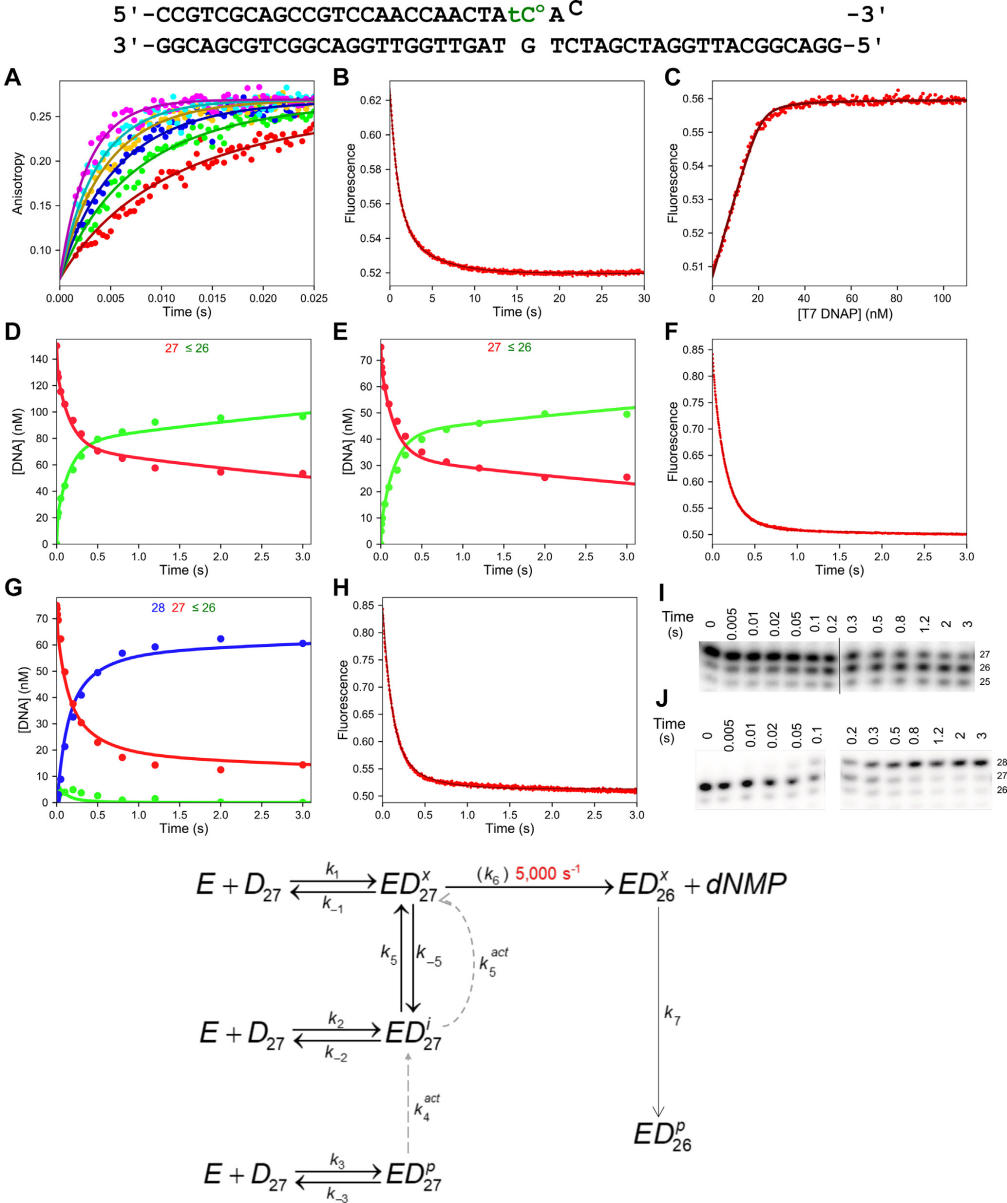
**Kinetics of binding and excision of a 3′ mismatch.** DNA substrate: The DNA substrate used in the experiments shown in this figure is given at the *top*. For experiments with FAM-labeled DNA, the DNA primer strand contained a 5’-[6-FAM] label, and the tC^o^ was a substituted with C in the primer. *Solid lines* through the data in this figure show the best fits by simulation using the kinetic scheme at the *bottom* of this figure with parameters listed in Table 3. For each experiment, we quote values obtained by equation-based data fitting, but we show only the curves derived by global data fitting. Experiments in (*A*) through (*C*) were performed to measure DNA binding using hydrolysis-resistant phosphorothioate (S_p_ isomer) 3′-terminal mismatch DNA. *A*, Stopped-flow 3′ mismatch DNA binding rate experiment. A solution of 25 nM FAM-labeled phosphorothioate 3-terminal mismatch DNA (FAM-27-PThio/45-3′MM-PThio) (12.5 mM Mg^2+^) was mixed with varying concentrations of T7 DNA polymerase (100–400 nM) and 2 μM thioredoxin to start the reaction. Measuring fluorescence anisotropy gives the net binding rate for all DNA binding sites on the enzyme, and this experiment estimates a net second-order binding rate constant around 1000 μM^−1^ s^−1^. *B*, stopped-flow 3′ mismatch DNA off-rate experiment. Fluorescence was monitored after mixing a solution of 25 nM enzyme–DNA complex (tC^o^ phosphorothioate DNA with a 3′-terminal mismatch [tC^o^-27-PThio/45-3′MM-PThio], 30 nM T7 DNA polymerase, 1 μM thioredoxin, 12.5 mM Mg^2+^) with 500 nM unlabeled trap DNA (phosphorothioate DNA with a 3′-terminal mismatch, 27-PThio/45-3′MM-PThio). The data best fit two exponentials with rates of 1.16 ± 0.02 s^−1^ and 0.270 ± 0.003 s^−1^, and 65% of the total amplitude occurred during the fast phase. *C*, equilibrium DNA binding titration experiment. A solution of 20 nM tC^o^ 3′ mismatch phosphorothioate DNA (tC^o^-27-PThio/45-3′MM-PThio) and 2 μM thioredoxin in a cuvette was titrated with T7 DNA polymerase with constant mixing from a micro star bar over the course of 5 min. The data were corrected for the small dilution and inverse inner filter effect, and the corrected data are shown. Conventional fitting of the experiment to a quadratic equation estimates a *K*_*d*_ for DNA binding of 0.7 ± 0.1 nM. *D*, rapid-quench 3′ mismatch DNA binding and excision experiment. A solution of 150 nM ^32^P-tC^o^ 3′ mismatch DNA (^32^P-tC^o^-27/45-3′MM) (12.5 mM Mg^2+^) was mixed with 1 μM T7 DNA polymerase, 10 μM thioredoxin, and 0.1 mg/ml BSA to start the reaction. Aliquots were quenched with EDTA at various times, products were resolved by denaturing PAGE, and the gel was visualized by phosphorimaging (shown in *I*). The concentration of 27 nt primer is shown in *red*, and the concentration of products less than or equal to 26 nt are shown in *green*. The data best fit three exponentials: a fast phase at >200 s^−1^ with an amplitude of 19 ± 2 nM, a second phase at 9 ± 5 s^−1^ with an amplitude of 26 ± 17 nM, and a third phase at 2.0 ± 0.6 s^−1^ with an amplitude of 52 ± 17 nM. *E*, rapid-quench Mg^2+^ addition to preincubated E–DNA complex experiment. A solution of 1 μM T7 DNA polymerase, 75 nM ^32^P-tC^o^ 3′ mismatch DNA (^32^P-tC^o^-27/45-3′MM), 10 μM thioredoxin, and 0.1 mg/ml BSA was mixed with 12.5 mM Mg^2+^ to start the reaction, and samples were processed as in (*D*). The data best fit three exponentials: a fast phase at 168 ± 59 s^−1^ with an amplitude of 7.1 ± 1.2 nM, a second phase at 7 ± 2 s^−1^ with an amplitude of 22 ± 6 nM, and a third phase at 1.53 ± 0.46 s^−1^ with an amplitude of 21 ± 6 nM. *F*, stopped-flow Mg^2+^ addition to preincubated E–DNA complex experiment. A solution of 100 nM T7 DNA polymerase, 2 μM thioredoxin, and 40 nM tC^o^-3′ mismatch DNA (tC^o^-27/45-3′MM) was mixed with 12.5 mM Mg^2+^ to start the reaction. The data best fit two exponentials, with the fast phase having 88% of the total amplitude at a rate of 7.24 ± 0.04 s^−1^ and the slow phase having a rate of 1.86 ± 0.05 s^−1^. *G*, rapid-quench 3′ mismatch excision–extension experiment. A solution of 75 nM ^32^P-tC^o^ 3′ mismatch DNA (^32^P-tC^o^-27/45-3′MM), 400 nM T7 DNA polymerase, 8 μM thioredoxin, 0.1 mg/ml BSA, and 1 mM EDTA was mixed with 12.5 mM Mg^2+^ and 1 μM unlabeled 3′ mismatch DNA as a trap, 100 μM dGTP, and 100 μM dATP to start the reaction. Aliquots were quenched with EDTA, processed as in (*D* and *E*)—the corresponding gel is shown in (*J*). Data corresponding to the 27, 26, and 28 nt products are colored in *red*, *green*, and *blue*, respectively. Data for the 27 nt DNA best fit a two-exponential function with rates of 8.3 ± 2.2 s^−1^ and 2.6 ± 0.73 s^−1^ with amplitudes of 30 ± 13 nM and 32 ± 13 nM for the fast and slow phases, respectively. *H*, stopped-flow 3′ mismatch excision and then polymerization experiment. A solution of 40 nM tC^o^ 3′ mismatch DNA (tC^o^-27/45-3′MM), 100 nM T7 DNA polymerase, 2 μM thioredoxin, and 1 mM EDTA was mixed with 12.5 mM Mg^2+^, 1 μM unlabeled 3′ mismatch DNA, 100 μM dGTP, and 100 μM dATP to start the reaction. Fitting the data to two exponentials gives a fast phase with 91% of the total amplitude at 7.63 ± 0.04 s^−1^ and the slow phase at an observed rate of 0.75 ± 0.04 s^−1^. *I*, gel for rapid-quench 3′ mismatch DNA binding and excision experiment. Gel from the experiment in (*D*). The *black vertical line* indicates where two gel images were spliced together to make the continuous gel figure. DNA product lengths are labeled to the *right* of the gel. *J*, gels for rapid-quench 3′ mismatch excision–extension experiment. Gels from experiment in (*G*). DNA product lengths are labeled to the *right* of the gel. The space between the lanes indicates that samples were run on two different gels and combined here for the figure. Scheme: Kinetic model for 3′ mismatch excision by T7 DNA polymerase. *Solid arrows* indicate rate constants defined by the experiments without dNTPs present. The *dashed lines* represent the rate constants that change when dNTPs are added to the reaction. The rate of chemistry (*k*_*6*_) was locked at 5000 s^−1^ based on results for ssDNA excision. BSA, bovine serum albumin; FAM, carboxyfluorescein; tC^o^, 1,3-diaza-2-oxophenoxazine.

Although ssDNA hydrolysis is important mechanistically and puts estimates on the rate of chemistry occurring at the exo active site, dsDNA containing mismatch(es) at the 3′-end of the primer strand in duplex DNA are mechanistically more important as the enzyme must sense the mismatch and repair it faithfully to improve DNA replication fidelity. Therefore, we investigated the kinetics of hydrolysis of a single 3′ mismatch, the product formed after the enzyme misincorporates a base during replication at the pol active site. To build the kinetic model from the ground up, we begin by performing experiments to measure binding using hydrolysis-resistant phosphorothioate dsDNA substrates labeled with FAM or tC^o^. First, we measured the DNA binding rate using fluorescence anisotropy with a double-stranded FAM phosphorothioate 3′ mismatch DNA substrate shown at the *top* in Figure 4. A solution of nonhydrolyzable FAM-DNA was mixed with varying concentrations of T7 DNA pol (with E in excess) to start the reaction in the stopped-flow, and the increase in anisotropy upon DNA binding was monitored (Fig. 4*A*). Initial inspection of the traces shows that, unlike for ssDNA, the traces all approach the same end point, suggesting DNA binding is tight relative to the fixed DNA concentration (25 nM). The binding rate is also fast, occurring on a 25 ms timescale for the concentrations used in the experiment, which suggests a net diffusion–limited DNA binding rate of ∼1000 μM^−1^ s^−1^, similar to the ssDNA binding kinetics. As we will show with subsequent experiments, the kinetic model for exo hydrolysis on dsDNA substrates is complex, requiring multiple techniques to monitor different aspects of the reaction to provide a full model derived by global data fitting. We initially performed a DNA binding rate experiment using the tC^o^ fluorescence with the phosphorothioate DNA substrate, but the signal was too complicated to interpret (data not shown). The advantage of anisotropy in this experiment is that the signal change is known to be due to DNA binding to the enzyme. Thus, this experiment defines the net binding rate for all sites on the enzyme, which is an important constraint, although this experiment does not provide information on the fraction that binds at each site (pol *versus* exo). Next, we used the tC^o^ phosphorothioate 3′ mismatch substrate to measure the DNA dissociation rate (Fig. 4*B*). The data best fit two exponentials, with rates of approximately 1.2 s^−1^ and 0.27 s^−1^, although interpretation of this experiment alone or with the binding data does not define the dissociation rate from each site. To include equilibrium constraints on the fitting, an equilibrium titration was performed with increasing concentrations of T7 DNA pol at a fixed concentration of tC^o^-labeled DNA (Fig. 4*C*). Compared with the ssDNA titration curve in Figure 2*C*, the 3′ mismatch dsDNA titration clearly shows that this substrate binds the T7 DNA pol with much higher affinity than ssDNA, and fitting to a quadratic equation estimates a net *K*_*d*_ of less than 1 nM. Because of the complex nature of the reaction with multiple DNA binding sites on the enzyme, even global fitting of the binding data was insufficient to produce a unique model. These experiments however provide important constraints on the rate constants for DNA binding and dissociation that, when fit with the excision data described later, is a prime example of a case where the whole is greater than the sum of the parts (19).

Next, we performed rapid-quench and stopped-flow assays on the hydrolyzable substrate containing a 3′ mismatch to accompany the binding data from the previous section. In the first experiment, we measured the rate of mismatch excision upon mixing the enzyme and DNA to start the reaction in the quench-flow (Fig. 4*D*). To ensure continuity between the stopped-flow and rapid-quench experiments, we opted to label the tC^o^ dsDNA substrate with a 5′-^32^P to use in the rapid-quench experiments. A solution of T7 DNA pol (consisting of the T7 gp5 catalytic subunit and the thioredoxin accessory protein) was mixed with ^32^P-tC^o^ 3′ mismatch dsDNA (^32^P-tC^o^-27/45-3′MM) to start the reaction in the quench-flow. Samples were quenched at various times with EDTA, and products were resolved by denaturing PAGE (Fig. 4*I*). Preliminary inspection of the gel shows that the enzyme removes the terminal mismatch relatively quickly and then much more slowly removes properly base-paired bases in the DNA duplex. Figure 4*D* shows the concentration of the different DNA species *versus* time for this experiment with best fits to three exponentials. Previous studies on this enzyme (2) only showed two exponentials for hydrolysis of similar substrates. Here, fitting the data by simulation afforded a rationale for three kinetically significant steps and provided a justification for the additional phase. The data best fit a model with three DNA binding sites on the enzyme. The high enzyme concentration used in our experiment (1 μM) relative to the concentration used previously (300 nM) (2) makes the second-order rate constant for binding directly at the exo active site faster (net DNA binding rate is known from anisotropy binding experiment), revealing the fraction that binds directly to the exo active site and is rapidly hydrolyzed. The slightly slower phase defines DNA binding to an intermediate partially melted state on the enzyme that is then transferred to the exo active site for hydrolysis. The slowest phase represents the fraction of DNA that binds to the pol active site, slowly dissociates, and rebinds at one of the other two sites on the enzyme before being hydrolyzed. The smooth curves in Figure 4 show the global data fitting according to the scheme and rate constants shown at the *bottom* of the figure. The flux calculation feature in KinTek Explorer, derived from partial derivatives computed by numerical integration, was used to determine the fraction of DNA that binds from solution at each site for this substrate (Fig. S2), showing that approximately 40% of the DNA binds at the pol active site, 47% binds at the intermediate site, and 12% binds directly to the exo active site.

Next, a similar rapid-quench experiment was performed, except the enzyme and DNA were preincubated in the absence of Mg^2+^, and then Mg^2+^ was then added to start the reaction. Therefore, the DNA pre-equilibrates between the different enzyme sites before the reaction is initiated with Mg^2+^. The results of this experiment are shown in Figure 4*E*. The data are almost identical to the experiment in Figure 4*D*, and fitting by simulation to the model in Figure 4 estimates that approximately 34%, 57%, and 8% of the DNA is in the pol, intermediate, and exo active sites, respectively, at equilibrium in the absence of Mg^2+^. These fractions are similar to the fraction that binds from solution at each site in the presence of Mg^2+^ and provide important constraints to interpret stopped-flow fluorescence signals where it is impossible to accurately determine the fraction in each site without this information. With these constraints, we performed the same Mg^2+^ addition to the preincubated enzyme–DNA complex experiment in the stopped-flow to monitor changes in hybridization state of the DNA upon Mg^2+^ addition (Fig. 4*F*). The decrease in fluorescence arises from the transfer of the trimmed primer strand back to the pol active site after mismatch excision. Conventional data fitting to equations estimate an observed rate of ∼7 s^−1^ for this step, but fitting by simulation reveals that the observed rate is limited by transfer to the exo active site before hydrolysis, and the rate constant for transfer to the pol active site after mismatch hydrolysis (*k*_*7*_ in the scheme in Fig. 4) is approximately 60 s^−1^.

Since the scaling factors in fluorescence studies are difficult to interpret without additional information, we performed further experiments where mismatches were excised in the presence of added nucleotides to allow extension of the primer after the mismatch is removed (Fig. 4, *G*, *H*, and *J*). After the mismatch is removed at the exo active site, the primer must transfer back to the pol active site before additional nucleotides can be added. Because polymerization is much faster than strand transfer, any fluorescence signal that correlated with the rate of primer extension could be attributed to the DNA transfer step. First, we used rapid-quench methods to monitor the progress of the reaction, where the enzyme–^32^P–tC^o^ 3′ mismatch dsDNA complex was mixed with Mg^2+^, dGTP, dATP, and an excess of unlabeled 3′ mismatch DNA as a trap to start the reaction (Fig. 4*G*). Time points were analyzed as before, and the resulting gel is shown in Figure 4*J*. As shown in Figure 4*G*, the 26 nt intermediate does not accumulate. Rather, the band for the 26 nt intermediate forms and decays rapidly as the enzyme extends the 26 nt primer by two bases to form the 28 nt product. These data support the notion that transfer from the exo active site to the pol active site after mismatch excision is fast (∼60 s^−1^) relative to the rate of excision.

Since a trap (unlabeled DNA) was included in the reaction, all 28 nt products that are formed results from excision of the mismatch, transfer to the pol site, and extension without dissociation from the enzyme. Note that the amplitude of product formation in the mismatch excision–extension experiment (Fig. 4*G*) is higher than the excision-only experiments (Fig. 4, *D* and *E*), even though a trap was included in the excision–extension experiment.

We will later show that the nucleotide-dependent increase in amplitude is even more pronounced for the buried mismatch substrate. To account for these observations based on the same base model as for the experiments without nucleotide, the model required modification to include a nucleotide-dependent transfer from the *ED*_*p*_ to the *ED*_*i*_ state (*k*_*4,act*_ in the scheme in Fig. 4) and a nucleotide-dependent increase in the rate of transfer from *ED*_*i*_ to *ED*_*x*_ (*k*_*5,act*_ in the scheme in Fig. 4). For this substrate, it is interesting that without dNTPs, there is no significant transfer from *ED*_*p*_ to *ED*_*i*_, but in the presence of dNTPs, the transfer occurs readily. Finally, we repeated the same experiment in the stopped-flow to monitor the rate of transfer from the exo active site to the pol active site after mismatch excision (Fig. 4*H*). Conventional analysis of the data in this figure shows that the observed rates are similar to those in the experiment in Figure 4*F*, albeit slightly faster. Furthermore, based on our model, the slow linear phase represents the small fraction of DNA that was bound to the ED_p_ state and is slowly transferred to the exo active site in the presence of dNTPs.

**Figure 5:**
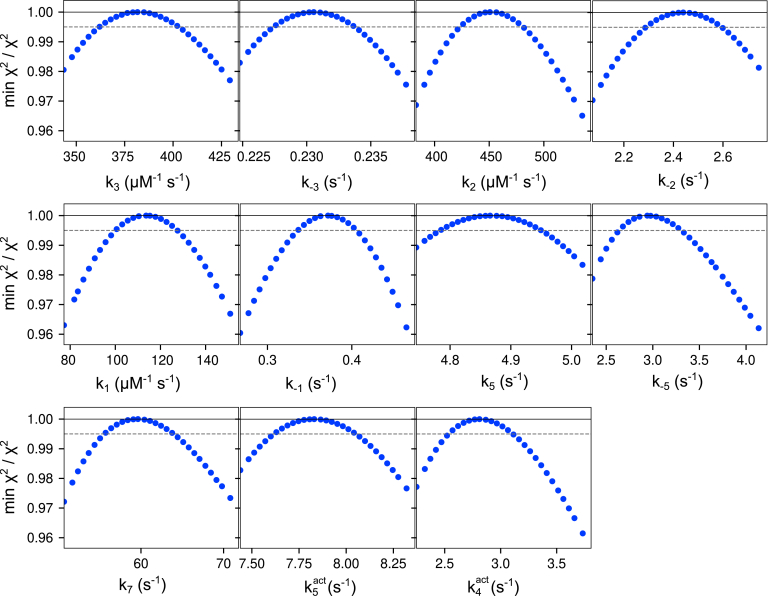
**Confidence contours for global fit of 3′ mismatch DNA.** Confidence contours were calculated with the FitSpace feature of KinTek Explorer. Note that *k*_*6*_ was locked at 5000 s^−1^ in the fitting. A 0.995% confidence threshold, shown as the *dashed gray line*, represents the 95% confidence interval, based on the number of data points and the number of parameters in the fitting, as calculated by the software. Best-fit parameters are given in Table 3.

Global data fitting by simulation in KinTek Explorer was essential to develop a model that accounted for all the experimental data shown in Figure 4 with rate constants listed in Table 3. Because of the large number of parameters in the complex model, it was critical to perform confidence contour analysis with the FitSpace function of KinTek Explorer to determine whether each parameter was supported by the data—the results are shown in Figure 5. Parabolic curves indicate that each parameter is well constrained by the data, including the “activated” steps where some rate constants are stimulated in the presence of dNTPs. The main features of the model include three binding sites for the DNA on the enzyme, one site being the exo active site, another being the pol active site, and the third being an intermediate with partially melted DNA. The identity of the intermediate state is unknown, but the fluorescence data show that it has a higher fluorescence than complexes at the pol active site, suggesting it is at least partially melted. Our model also includes the fast rate of chemistry (∼5000 s^−1^) estimated from the ssDNA experiments. Finally, in the presence of nucleotides, the kinetics of transfer between the sites change. In the absence of dNTPs, there is no evidence for direct transfer from the pol active site to the exo active site. However, in the presence of nucleotides, data fitting requires a transfer step from the pol to the intermediate state and a nucleotide-dependent stimulation of the rate of transfer from the intermediate state to the exo active site. Unfortunately, we cannot address whether the nucleotide-dependent transfer rate requires proper base pairing with the template strand because our method of measurement requires fast template-dependent extension of the 26 nt product of mismatch excision. With a model for excision of the 3′ mismatch substrate, we next investigated the kinetics of excision on a mismatch buried by a correct base pair.

**Table 3:** Kinetic parameters for mismatched DNA^a^

Parameter	Best fit	95% Confidence interval
*k*_*1*_	114 μM^−1^ s^−1^	(101, 125)
*k*_*-1*_	0.372 s^−1^	(0.337, 0.402)
*k*_*2*_	451 μM^−1^ s^−1^	(426, 477)
*k*_*-2*_	2.44 s^−1^	(2.31, 2.58)
*k*_*3*_	382 μM^−1^ s^−1^	(362, 402)
*k*_*-3*_	0.231 s^−1^	(0.228, 0.234)
*k*_*5*_	4.87 s^−1^	(4.79, 4.95)
*k*_*-5*_	2.94 s^−1^	(2.68, 3.27)
*k*_*6*_	**5000 s^−1^**	Locked
*k*_*7*_	59.6 s^−1^	(55.7, 63.8)
*k*_*4,act*_	2.81 s^−1^	(2.57, 3.07)
*k*_*5,act*_	7.83 s^−1^	(7.63, 8.04)

a Rate constants derived in Figure 4 are summarized with confidence intervals derived by contour analysis. Because the contours are symmetrical, we can estimate the standard errors directly from the confidence intervals, for example, *k*_*1*_ = 114 ± 12 μM^−1^ s^−1^ with lower and upper limits of 101 and 125. The value of *k*_*6*_ = 5000 s^−1^ (shown in *bold text*) for the rate of hydrolysis of the 3′-terminal nucleotide was locked at a lower limit sufficient to account for the observed rates.

### Kinetics of buried mismatch excision

**Figure 6:**
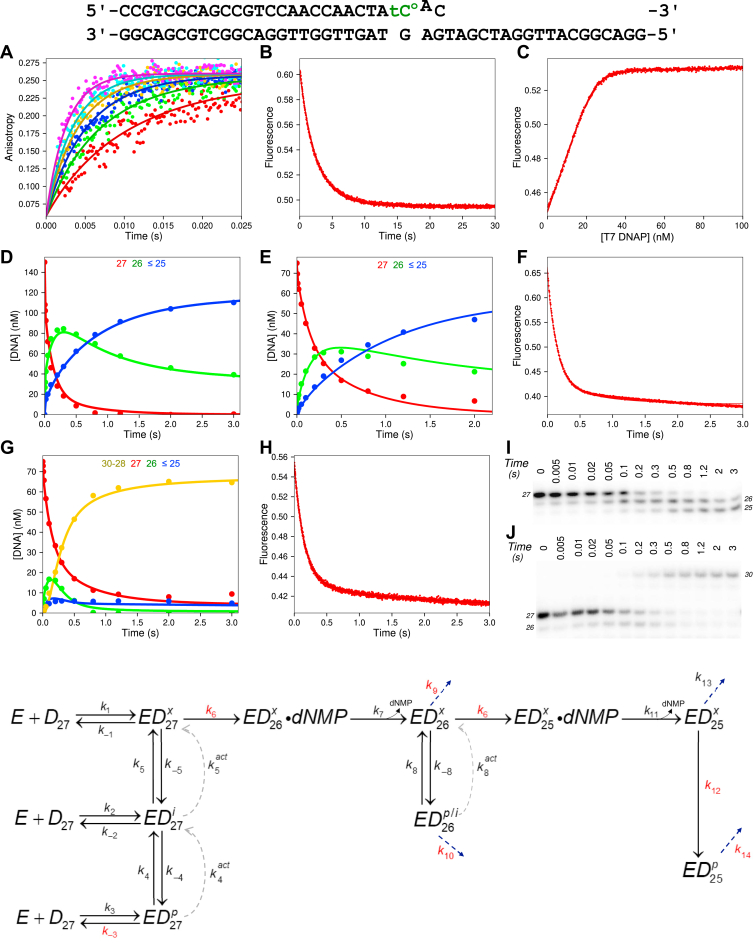
**Buried mismatch binding and excision kinetics.** DNA substrate: The DNA substrate used in these experiments is shown at the *top* of the figure. For experiments with FAM-labeled DNA, the location given for tC^o^ in the substrate was changed to a C and the primer contained a 5′-6-FAM label. Experiments in (*A*) through (*C*) were performed with hydrolysis-resistant phosphorothioate (S_p_ isomer) DNA to measure binding. In all experiments, the *solid line* through the data shows the best global fit by simulation in KinTek Explorer using the model at the *bottom* of the figure. *A*, stopped-flow buried mismatch DNA binding-rate measurement. A solution of 25 nM FAM-buried mismatch phosphorothioate DNA (FAM-27-PThio/45-BMM-PThio) and 12.5 mM Mg^2+^ was mixed with 2 μM thioredoxin and varying concentrations of T7 DNA polymerase (100–400 nM) to start the reaction. The data fitting provided an estimate a net second-order DNA binding rate constant of approximately 1000 μM^−1^ s^−1^. *B*, stopped-flow buried mismatch DNA off-rate experiment. A solution of 25 nM tC^o^-buried mismatch phosphorothioate DNA (tC^o^-27-PThio/45-BMM-PThio), 30 nM T7 DNA polymerase, 600 nM thioredoxin, and 12.5 mM Mg^2+^ was mixed with 500 nM unlabeled buried mismatch phosphorothioate DNA (27-PThio/45-BMM-PThio) to start the reaction. The data fit two exponentials with rates of 0.60 ± 0.02 s^−1^ and 0.25 ± 0.01 s^−1^ with 60% of the total amplitude corresponding to the fast phase. *C*, equilibrium buried mismatch DNA binding titration. A solution of 25 nM tC^o^ buried mismatch phosphorothioate DNA (tC^o^-27-PThio/45-BMM-PThio), 2 μM thioredoxin, and 12.5 mM Mg^2+^ in a cuvette was titrated with T7 DNA polymerase with constant mixing from a micro stir bar. The signal was corrected for the small dilution, and the inverse inner filter effect and the corrected data are shown. Fitting the titration curve to a quadratic equation estimates a *K*_*d*_ of approximately 0.5 nM. *D*, rapid-quench buried mismatch binding and excision experiment. A solution of 150 nM ^32^P-tC^o^ buried mismatch DNA (^32^P-tC^o^-27/45-BMM) and 12.5 mM Mg^2+^ was mixed with 1 μM T7 DNA polymerase, 10 μM thioredoxin, and 0.1 mg/ml BSA to start the reaction. Time points were quenched with EDTA, separated by denaturing PAGE, and visualized by phosphorimaging. The gel is shown in (*I*). Products of 27, 26, and ≤25 nt in length are colored in *red*, *green*, and *blue*, respectively. The data for the loss of 27 nt DNA best fit two exponentials with a fast phase with an amplitude of 45 ± 3 nM at 350 ± 115 s^−1^ and a slow phase with an amplitude of 105 ± 4 nM at a rate of 7.3 ± 0.6 s^−1^. *E*, rapid-quench Mg^2+^ addition to preincubated E–DNA complex. A solution of 75 nM ^32^P-tC^o^ buried mismatch DNA (^32^P-tC^o^-27/45-BMM), 1 μM T7 DNA polymerase, 10 μM thioredoxin, 0.1 mg/ml BSA, and 1 mM EDTA was mixed with 12.5 mM Mg^2+^ to start the reaction. Samples were processed as in (*D*). Products of 27, 26, and ≤25 nt in length are colored in *red*, *green*, and *blue*, respectively. The data for the loss of the 27 nt DNA best fit a double exponential function. The fast phase has an amplitude of 12 ± 2 nM at a rate of 84 ± 31 s^−1^ and a slower phase with an amplitude of 56 ± 2 nM and a rate of 3.5 ± 0.3 s^−1^. *F*, stopped-flow Mg^2+^ addition to preincubated E–DNA complex. A solution of 40 nM tC^o^-buried mismatch DNA (tC^o^-27/45-BMM), 100 nM T7 DNA polymerase, 2 μM thioredoxin, and 1 mM EDTA was mixed with 12.5 mM Mg^2+^ to start the reaction. The data best fit two exponentials with a fast phase with 82% of the total amplitude at 6.73 ± 0.03 s^−1^ and a slower phase at 0.35 ± 0.02 s^−1^. *G*, rapid-quench buried mismatch excision–extension experiment. A solution of 75 nM ^32^P-tC^o^ buried mismatch DNA (^32^P-tC^o^-27/45-BMM), 400 nM T7 DNA polymerase, 8 μM thioredoxin, 0.1 mg/ml BSA, and 1 mM EDTA was mixed with 1 μM unlabeled buried mismatch DNA as a trap, 100 μM dATP, 100 μM dCTP, 100 μM dTTP, and 12.5 mM Mg^2+^ to start the reaction. Samples were processed as in (*D* and *E*), and the gel is shown in (*J*). Products of 30, 27, 26, and ≤25 nt in length are shown in *yellow*, *red*, *green*, and *blue*, respectively. Data for the loss of the 27 nt DNA best fit two exponentials with a fast phase with an amplitude of 18 ± 4 nM at a rate of 19 ± 5 s^−1^ and a slower phase with a rate of 3.5 ± 0.3 s^−1^ and an amplitude of 48 ± 4 nM. *H*, stopped-flow buried mismatch excision–extension experiment. A solution of 25 nM tC^o^ buried mismatch DNA (tC^o^-27/45-BMM), 40 nM T7 DNA polymerase, and 800 nM thioredoxin was mixed with 600 nM unlabeled buried mismatch DNA as a trap, 100 μM dATP, 100 μM dCTP, 100 μM dTTP, and 12.5 mM Mg^2+^ to start the reaction. The data best fit two exponentials with a fast phase at a rate of 6.66 ± 0.04 s^−1^ with 82% of the total amplitude and a slower phase at 0.50 ± 0.03 s^−1^. *I*, gel for rapid-quench buried mismatch binding and excision experiment. Gel from the experiment in (*D*). DNA product lengths are labeled to the *left* and *right* of the gel. *J*, gel for rapid-quench buried mismatch excision–extension experiment. Gel from the experiment in (*G*). DNA product lengths are labeled to the *left* and *right* of the gel. Scheme: Kinetic model for buried mismatch excision. Rate constants locked during the fitting are shown in *red*. *Dashed dark blue arrows* indicate states from which the DNA can dissociate from the enzyme. These dissociation rate constants were mostly locked at values obtained for the 3′ mismatch substrate. BSA, bovine serum albumin; FAM, carboxyfluorescein; tC^o^, 1,3-diaza-2-oxophenoxazine.

In our previous article on substrate specificity for the exo proofreading domain of T7 DNA pol (11), we showed that buried mismatches are excised much more rapidly than single 3′’ mismatches. This reaction provides an additional mechanism for error correction by first extending the mismatch with the correct base before highly efficient excision of two nucleotides including the mismatch and the added nucleotide. Here we investigate the detailed kinetics governing how the buried mismatch is more efficiently hydrolyzed than the single 3’ mismatch substrate. As for the other substrates investigated in this paper, we begin with binding measurements using the hydrolysis-resistant phosphorothioate oligonucleotide substrates. First, we measured the net DNA binding rate using fluorescence anisotropy by stopped-flow after mixing a solution of FAM phosphorothioate buried mismatch DNA (FAM-27-PThio/45-BMM-PThio) with varying concentrations of T7 DNA polymerase and thioredoxin (Fig. 6*A*). As for the 3’ mismatch substrate, the net DNA binding rate was fast, approaching a second-order rate constant of 1,000 μM^−1^ s^−1^. All traces approach the same endpoint, indicating tight binding relative to the fixed DNA concentration (25 nM). As aforementioned, this experiment does not define individual rate constants by itself because it represents the sum of binding to two or three enzyme sites but provides important constraints on the net binding rate for the sum of all DNA binding sites on the enzyme. We next measured the DNA dissociation rate in the stopped-flow by mixing a preincubated solution of tC^o^ phosphorothioate buried mismatch DNA (tC^o^-27-PThio/45-BMM-PThio) and T7 DNA pol with a large excess of unlabeled phosphorothioate buried mismatch DNA (27-PThio/45-BMM-PThio) as a trap for free enzyme that has dissociated from the labeled DNA. This experiment provides important constraints that become meaningful when globally fit with the other experiments in Figure 6. We next performed an equilibrium titration experiment by titrating the T7 DNA pol complex into a solution of tC^o^ phosphorothioate buried mismatch DNA over the course of 5 min with constant mixing from a micro stir bar—the results are shown in Figure 6*C*. Fitting the titration curve to a quadratic equation provides a net *K*_*d*_ of approximately 0.5 nM, providing an additional equilibrium constraint on the binding parameters. Also, this experiment demonstrates that even though the DNA contains mismatches, the *K*_*d*_ is similar to the *K*_*d*_ for DNA containing a single mismatch, suggesting the binding is not weaker; however, the partitioning of the DNA between sites is different, as described later.

Next, we measured the kinetics of buried mismatch excision using experiments similar to those performed for the 3′ mismatch substrate. In the first rapid-quench experiment, a solution of T7 DNA pol was mixed with ^32^P-labeled tC^o^ buried mismatch DNA (^32^P-tC^o^-27/45-BMM) to start the reaction, and samples were collected and processed as described for the 3′ mismatch experiments and shown in Figure 6*D*. The data for the loss of the 27 nt starting material best fit at least two exponentials, with a very fast phase that corresponds to the fraction of DNA that binds directly to the exo active site from solution and is rapidly hydrolyzed, followed by a slower phase that likely represents transfer of the primer strand from another site on the enzyme to the exo active site. Relative to the 3′ mismatch substrate, the amplitude of the fast phase is greater and the rate of the slower phase is faster, supporting the model where the buried mismatch has more single-stranded character and favors partitioning and transfer into the exo active site. The gel for this experiment is shown in Figure 6*I* and shows that the enzyme removes the terminal two bases of the primer strand and then pauses and very slowly excises the remaining correctly base-paired nucleotides of the primer strand. Flux calculations (Fig. S3) estimate that approximately 2%, 64%, and 34% of the DNA binds from solution at the pol active site, intermediate site, and exo active site, respectively. Next, we performed the experiment where ^32^P-tC^o^ buried mismatch DNA, preincubated with T7 DNA pol, was mixed with Mg^2+^ to start the reaction in the quench-flow (Fig. 4*E*). In this experiment, global data fitting indicates that when preincubated in the absence of Mg^2+^, approximately 27%, 64%, and 9% of the DNA occupies the pol active site, intermediate site, and exo active site, respectively. In our preliminary studies, we discovered that Mg^2+^ has a large effect on the binding and partitioning kinetics for various substrates, and the fractions in each site in the absence of Mg^2+^ *versus* the fraction that binds from solution for this experiment varies greatly (data not shown). Because Mg^2+^ has large effects on DNA binding (20), the fraction of DNA in each site in the absence of Mg^2+^ may not be physiologically relevant. However, this experiment provides important constraints for the next stopped-flow experiment that provides additional information on the rates of transfer between sites. To monitor the kinetics of transfer to the pol active site after Mg^2+^ addition and mismatch excision, we performed a stopped-flow experiment where a solution of tC^o^ buried mismatch DNA (tC^o^-27/45-BMM) and T7 DNA pol was mixed with Mg^2+^ to start the reaction (Fig. 6*F*). The data are biphasic, with 82% of the total amplitude at 6.73 ± 0.03 s^−1^ and a slower phase observed at 0.35 ± 0.02 s^−1^.

To provide additional data to interpret the fluorescence signal from the tC^o^ label, we performed excision–extension experiments, as were performed previously for the 3′ mismatch substrate. First, we performed an experiment by rapid-quench to define the kinetics of DNA excision and extension (Fig. 6*G*). A solution of T7 DNA pol and ^32^P-tC^o^ buried mismatch DNA was mixed with Mg^2+^, dATP, dCTP, dTTP, and an excess of unlabeled buried mismatch DNA as a trap to start the reaction. Samples were processed as described previously, and the resulting gel is shown in Figure 6*J*. From the gel, we see rapid excision of the terminal base, but little accumulation of the 25 nt intermediate, indicating that as soon as the 3′-terminal base is excised, the primer partitions back into the pol active site, and nucleotides are rapidly added to the primer strand to form the 30 nt product. When comparing this experiment in Figure 6*G* to the similar experiment in Figure 6*E*, it is clear that the excision kinetics change significantly in the presence of dNTPs. Since the experiment was performed in the presence of excess unlabeled DNA, all observed excision kinetics occur within a single binding event. When looking at the rate of loss of the starting 27 nt primer, the rate is visibly faster than the corresponding curve in Figure 6*E*. Furthermore, the 26 nt product is much more rapidly hydrolyzed to the 25 nt product, whereas without dNTPs, the 26 nt product is slowly trimmed to the 25 nt product. We then performed a similar experiment in the stopped-flow by mixing a solution of T7 DNA pol and tC^o^ buried mismatch DNA with Mg^2+^, dATP, dCTP, dTTP, and an excess of unlabeled buried mismatch DNA as a trap to start the reaction (Fig. 6*H*). The observed rate is slightly faster than the experiment in Figure 6*F* without dNTPs; however, the rate of transfer from the exo to pol active site determined from data fitting does not appear to change.

**Figure 7:**
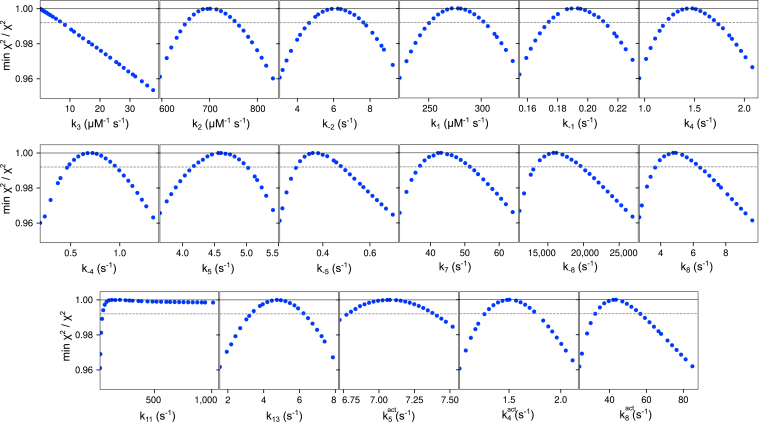
**Confidence contours for buried mismatch data.** Confidence contours were calculated with the FitSpace feature of KinTek Explorer. A threshold of 0.99 in the normalized χ^2^ ratio, shown as the *dashed gray line*, represents the 95% confidence interval, calculated using the F-distribution based on the number of data points and the number of parameters in the fitting as calculated by the software.

As for the other substrates, global data fitting by simulation was crucial to make sense of the data and is another example of a case where the whole is greater than the sum of the parts. The final model used for data fitting is shown in the scheme in Figure 6. There can be no question that the model accounts for the data based on the goodness of fit. The remaining question is whether the model is too complex, which we address by confidence contour analysis to assess the extent to which each parameter is well constrained by the data. Confidence contours in Figure 7 demonstrate that fitted parameters are well constrained by the data with well-defined upper and lower limits, except for two parameters, which only have lower or upper limits. It is understandable that there are no data to define the rate of dNMP release (*k*_*11*_ > 100 s^−1^). Similarly, the data only define an upper limit for DNA binding to the pol site (*k*_*3*_ < 6.7 μM^−1^ s^−1^). In addition, several rate constants were locked, based on preliminary fitting attempts, revealing that only a lower limit could be set for each of these constants, which are given in *red text* in the scheme in Figure 6 and Table 4. Despite the complexity of the model, the quality of the global fit and the significant number of well-constrained parameters gives confidence in the validity of the model to represent the reactions governing DNA partitioning between exo and pol sites.

**Table 4:** Buried mismatch kinetic parameters for buried mismatch excision^a^

Parameter	Best fit	95% Confidence interval
*k*_*1*_	278 μM^−1^ s^−1^	(268, 285)
*k*_*-1*_	0.193 s^−1^	(0.186, 0.196)
*k*_*2*_	701 μM^−1^ s^−1^	(686, 714)
*k*_*-2*_	6.23 s^−1^	(5.65, 6.5)
*k*_*3*_	6.66 μM^−1^ s^−1^	<6.66
*k*_*-3*_	**0.2 s**^**−1**^	Locked
*k*_*4*_	1.48 s^−1^	(1.38, 1.51)
*k*_*-4*_	0.722 s^−1^	(0.627, 0.777)
*k*_*5*_	4.56 s^−1^	(4.46, 4.71)
*k*_*-5*_	0.357 s^−1^	(0.342, 0.392)
*k*_*6*_	**5000 s^−1^**	Locked
*k*_*7*_	43.1 s^−1^	(41.9, 44.5)
*k*_*8*_	4.93 s^−1^	(4.49, 5.16)
*k*_*-8*_	16,200 s^−1^	(15,100, 16,700)
*k*_*9*_	**0.372 s**^**−1**^	Locked
*k*_*10*_	**2.44 s**^**−1**^	Locked
*k*_*11*_	101 s^−1^	>101
*k*_*12*_	**60 s**^**−1**^	Locked
*k*_*13*_	4.74 s^−1^	(4.46, 5.21)
*k*_*14*_	**0.2 s**^**−1**^	Locked
*k*_*4,act*_	1.5 s^−1^	(1.44, 1.57)
*k*_*5,act*_	7.08 s^−1^	(6.99, 7.15)
*k*_*8,act*_	43.5 s^−1^	(40.4, 44.6)

a Rate constants derived from fitting the data in Figure 6 are summarized with confidence intervals derived by contour analysis, with lower and upper limits given in parentheses. Parameters in *bold text* were locked during final fitting base on analysis showing that these rate constants had only lower limits in preliminary data fitting.

The main features of our model for the buried mismatch DNA substrate are as follows: just like for the 3′ mismatch DNA substrate, the best fit model contains three DNA binding sites on the enzyme, the pol active site, the exo active site, and a partially melted intermediate state. Unlike the 3′ mismatch substrate, transfer from the pol active site to the intermediate state readily occurs with this substrate, although only a small fraction binds directly into the pol active site. Interestingly, we had to add a rate-limiting dNMP release step following hydrolysis of the first base, following fast excision at 5000 s^−1^. In the absence of nucleotides, most of the DNA partitions out of the exo active site before hydrolysis of the second base of the primer strand, as demonstrated by the slow rates of excision of bases after the first, shown in Figure 6, *D* and *E*. In contrast, in the presence of nucleotides, the majority of the DNA partitions to remove the second base after excision of the first and almost the full amplitude of the starting material is hydrolyzed and then extended in a single turnover (in the presence of a large excess of unlabeled DNA as a trap). Both in the presence and absence of nucleotides, dNMP release after the second base is hydrolyzed is fast, only having a lower limit in the confidence contour analysis. This is consistent with the data for the 3′ mismatch, where the data show that chemistry and dNMP release appear to occur simultaneously.

## Discussion

The observed kinetics of DNA binding and excision are complex because the DNA can bind to either the exo or pol sites and can partition between the two sites. In the exo site, the 3′-terminal nucleotide of the DNA can be rapidly excised and then the 3′ terminus can return to the pol site for continued polymerization. After binding at the pol site, the DNA can be extended by polymerization or slip into the exo site to be shortened. Any one measurement attempting to address the pathway of base excision is a complex function of all the alternate pathways, and for this reason, no one experiment can be interpreted unambiguously. However, global data fitting can resolve a single unifying mechanism by including information from multiple experiments performed with different starting states and monitoring either fluorescence or chemical conversion steps (9, 19). In these studies, each experiment not only provides unique information but also lacks specific details that are filled in by parallel experiments. For example, it is often asserted that fluorescence signals are difficult to interpret because one does not know *a priori* what step in a reaction pathway produces a change in fluorescence, and the scaling factors relating signal intensity to concentration are unknown. Analysis of the concentration dependence resolves signals associated with binding steps, whereas measurements of the rates of the chemical reaction can be included to aid in understanding observable fluorescence signals. Globally fitting all the experiments resolves a single model to account for all the available data, and confidence contour analysis provides quantitative data to address whether the model is fully supported by the data (9, 19, 21, 22).

In this article, we have given a detailed kinetic analysis of proofreading for a high-fidelity DNA pol on three physiologically relevant substrates. We introduced a fluorescent DNA base analog tC^o^, which provided a large fluorescence signal upon DNA binding to the enzyme and from DNA melting required for binding in the exo active site. Careful selection of the sequence surrounding tC^o^ (12) and its position resulted in a DNA substrate that we expect to be much less perturbing of DNA structure than observed in similar studies using 2-aminopurine (23, 24, 25, 26, 27, 28, 29, 30). In some studies, we wanted to measure DNA binding to the exo site without complications from the subsequent chemistry step. Here, there are three alternatives: mutate the enzyme active site, alter the substrate, or omit Mg^2+^, which is essential for catalysis. Experiments to restrict exo activity by removing Mg^2+^ can be difficult to interpret because we know that Mg^2+^ plays an important role in shielding the negative charge on the DNA backbone to attenuate protein–DNA interactions. Accordingly, Mg^2+^ has been implicated in significantly changing the activity of many nucleic acid enzymes (31, 32, 33, 34). For example, studies using HIV reverse transcriptase kinetics revealed that first Mg^2+^ ion enters the active site chelated by the incoming nucleotide, whereas the second Mg^2+^ binds after the nucleotide-induced conformational change to facilitate catalysis (20). This study also included molecular dynamics simulations to illustrate the dynamic role of Mg^2+^ in electrostatic shielding the phosphates on the DNA backbone with local concentrations of ∼4 M. For T4 DNA pol, a study showed that Mg^2+^ was a key thermodynamic switch, and that in the absence of Mg^2+^, a hydrophobic exo site is preferred over the pol site for binding the primer terminus (7). In contrast, exo -deficient mutants and pol -switching mutants shift this equilibrium toward the pol active site. For these reasons, it was important for us to avoid using active site mutants in T7 DNA pol to study oligo binding the presence of Mg^2+^ or to chelate Mg^2+^ to allow binding but not chemistry. We opted to create hydrolysis-resistant substrates by developing methods to enzymatically degrade the R_p_ isomer of a racemic mixture of phosphorothioate oligonucleotides and then purifying the hydrolysis-resistant S_p_ isomer. These sulfur-containing oligos have been useful for mechanistic studies on other enzymes as well as in biotechnology (16, 17, 35, 36, 37). Here, they provide the ability to perform kinetic measurements to isolate the binding steps in the presence of Mg^2+^ without DNA cleavage. We believe that the phosphorothioate-containing substrates offer the best alternative to prevent chemistry when measuring binding to the exo site.

We began our kinetic studies in this article using an ssDNA substrate since it can bind directly into the exo active site without first undergoing a melting step as with dsDNA (2). First, we measured binding by fluorescence anisotropy with FAM-labeled DNA using the hydrolysis-resistant phosphorothioate ssDNA to provide an estimate of the second-order rate constant for ssDNA binding of 1100 μM^−1^ s^−1^. While this second-order rate constant is faster than some association constants measured for enzyme-nucleic binding (38, 39), it is within the diffusion limit (40) and can be rationalized by considering the high concentration of negative charge in the DNA backbone and the highly positive DNA binding clefts on the pol (39, 41). For comparison, the second-order rate constant for the binding of ssDNA to the ssDNA binding protein ranges from 1000 to 2000 μM^−1^ s^−1^ for *E. coli* and the human mitochondrial proteins, respectively (42, 43). We then measured the dissociation rate by competition to provide an estimate of 44 s^−1^, resulting in a *K*_*d*_ for ssDNA binding of 40 nM (Fig. 2 and Table 2). Subsequent studies with normal (no phosphorothioate) DNA supported the fast-binding rate and in addition set a lower limit of 5000 s^−1^ for the rate of the chemistry step, to account for the observed rate that appeared coincident with by the rate of diffusion-limited ssDNA binding. The lower limit for the rate of chemistry was set by global data fitting and confidence contour analysis of the DNA cleavage kinetics (Figs. 2 and 3). Because the observed rates of ssDNA cleavage are orders of magnitude faster than seen with duplex DNA, our kinetic analysis with ssDNA is used to define the rate of cleavage of an ssDNA segment melted from duplex DNA after it enters the exo active site. Analysis of the forward excision reaction in the presence of dCMP product indicated that the reaction is almost completely irreversible because of weak binding (and fast release) of the dCMP product at the exo active site but afforded an estimate of the equilibrium for the chemistry step at the active site (*K*_*2*_ = 3) favoring product formation. The majority of inhibition observed in this experiment (Fig. 2*F*) came from dCMP competing with DNA binding at the exo active site of the free enzyme, which was resolved in our global data fitting.

Next, we examined the detailed kinetics of hydrolysis of a single 3′ mismatched DNA substrate, which would form if the pol made a mistake (at the pol active site) during DNA replication. For an exo -deficient T7 DNA pol variant, we previously estimated misincorporation to occur somewhere on the order of one in a million nucleotides (9, 10), which is in line with estimates for other high-fidelity DNA polymerases (3). Outstanding questions in the literature ask how efficiently the mismatch is excised *versus* extended, whether the primer strand gets to the exo active site by direct transfer from the pol active site or by dissociation from the enzyme and rebinding at the exo active site, and, finally, whether the DNA directly partitions back to the pol active site after mismatch excision without dissociating from the enzyme (6, 7, 30, 44, 45). To address these questions, we measured the complete kinetic pathway for 3′ mismatch hydrolysis using both rapid-quench experiments and fluorescence experiments with DNA containing the fluorescent cytosine analog, tC^o^. All the DNA binding and cleavage reactions are kinetically linked and cannot be deconvoluted by any single measurement. Therefore, global data fitting was essential to resolve individual steps in the pathway, which led to a model where the DNA can bind to three distinct sites on the enzyme (Fig. 4 and Table 3). First is the exo active site, where the 3-terminal nucleotide of mispaired DNA (or ssDNA) is rapidly hydrolyzed and exhibits a characteristic high fluorescence state in studies with tC^o^ because of the melting of dsDNA to allow access into the exo active site. A second site is the pol active site, where nucleotides can be rapidly added (300–500 s^−1^) and has a low fluorescence state in studies with tC^o^. The third is an intermediate site for which the structural identity is not known but has an intermediate fluorescence suggesting the DNA is at least partially melted and is an important kinetically defined intermediate in the partitioning of DNA between the exo and pol active sites. Interestingly, in a study on exo proofreading for T4 DNA pol, the authors also suggested an intermediate state where the primer strand is partly denatured (46); therefore, the presence of such an intermediate may be generally applicable to DNA polymerases with a proofreading function. The presence of an intermediate state is consistent with the large (>25 Å) distance between the pol and exo active sites, the required melting of at least three base pairs, coupled to the rotation of the DNA duplex to reach from the pol to the exo site as implied by molecular dynamics simulations (11).

We previously showed that after a mismatch is incorporated at the pol active site, the next correct base can be added on top of the mismatch, effectively burying the mismatch, although this occurs at a slow rate (10). However, further extension by the addition of a second nucleotide on top of the buried mismatch is severely inhibited, increasing the likelihood that the pol will correct the mistakes before continued DNA synthesis (11). We also showed that buried mismatches are excised more efficiently than mismatches at the 3′-primer terminus. Faster excision of buried mismatch provides an additional opportunity to correct the mistake by first extending the mismatch and thereby enhancing the net DNA replication fidelity. A similar phenomenon has been shown for the human mitochondrial pol γ, which removes buried mismatches faster than single 3′-terminal mismatches (6), so this observation is likely not unique to T7 DNA pol. We therefore performed detailed kinetic experiments to measure the rate constants governing buried mismatch excision by T7 DNA pol. The rate constants tell the story behind why the buried mismatch is more efficiently excised. When binding from solution, almost no buried mismatch substrate binds at the pol active site. In contrast, almost a third of DNA with a 3′ mismatch binds at the pol site, whereas for the buried mismatch, the majority binds at the exo and intermediate sites. Once bound to the enzyme, the equilibrium constant for transfer of the buried mismatch primer from the pol site to intermediate state increases (K_4_ ∼ 2), and the equilibrium constant for the transfer from the intermediate state to the exo active site is around five times greater than for the 3′ mismatch substrate. For both DNA substrates, after excision of the mismatch, our data show that the trimmed primer is transferred back to the pol active site rapidly (60 s^−1^), without dissociating from the enzyme.

When we investigated the kinetics of excision in the presence of nucleotides to allow for polymerization following mismatch excision, we noticed that the rates and amplitudes of the curves changed showing that excision was even more efficient in the presence of added nucleotides. These experiments were performed in the presence of a trap for free enzyme (large excess of unlabeled DNA), indicating that the difference was not because of faster dissociation of the DNA and rebinding at the exo active site but rather direct transfer between sites on the enzyme without dissociation of the DNA from the enzyme. This phenomenon is not unique to T7 DNA pol as it has been shown for human DNA pol γ, where addition of nucleotide to the reaction that is identical to the 3′ base increased the rate of excision sevenfold. The authors proposed that the free nucleotide may enhance the rate of transfer of the DNA to the exo active site by interrupting the correct 3′ base pairing through interaction with the template base (6). This phenomenon has also been shown for Klenow fragment of *E. coli* DNA pol I where the authors used single-molecule FRET to measure the kinetics of site switching. The authors showed that dNTPs specifically accelerate pol to exo switching of a mispaired DNA terminus using active site mutants to prevent hydrolysis (5), but Klenow has a very inefficient exo reaction that may not relate to high-fidelity polymerases.

The structural basis for stimulation of the rate of transfer by free nucleotide remains unknown but poses an interesting question for structural or computational studies. Future studies could also be designed to measure the concentration dependence of nucleotide on the rates of switching to further understand the proofreading pathway for T7 DNA pol under physiological conditions or conditions of nucleotide starvation. To account for the different kinetics in the presence of nucleotides, we added “activation” steps to our pathways that occur in the presence of nucleotide. For the 3′ mismatch substrate, this had the effect of increasing the rate of transfer from the pol to intermediate site from close to 0 to over 2 s^−1^ and increasing the rate of transfer from the intermediate state to the exo active site by almost a factor of 2. For the buried mismatch substrate, the already appreciable rate of transfer from the pol to intermediate site was increased slightly, along with the already fast rate of transfer from the intermediate site to the exo site. The bigger effect however was to greatly reduce the rate of dissociation following excision of the terminal base, resulting in much faster hydrolysis of the subsequent mismatched base followed by transfer back to the pol site for continued polymerization.

With all the data combined, the answer to the question regarding direct pol to exo transfer *versus* dissociation and rebinding becomes more complicated. In the absence of nucleotides, our data suggest that the preferred route is through dissociation and rebinding, at least for the 3′ mismatch substrate. However, in the presence of nucleotides, the direct transfer pathway becomes more efficient with over 90% going through the direct transfer pathway. For the buried mismatch, direct transfer to the exo site is the favored pathway in the absence of nucleotides; however, the enzyme is most likely to remove only one base and then dissociates from the DNA, rebinds at the exo site, and then removes the next base. The presence of nucleotides shifts the equilibrium after excision of the first base toward excision of the mismatched base allowing proofreading of both bases in a single binding event. For both substrates in the presence and absence of nucleotides, after mismatch excision, the DNA is rapidly transferred back to the pol active site without dissociation. This can be rationalized structurally by favorable energetics of reannealing of the melted primer strand with the template strand. The results presented in this article define the kinetic pathway for proofreading for T7 DNA pol, and the overall themes are likely applicable to other high-fidelity replicative DNA polymerases.

## Experimental procedures

### Enzymes, oligonucleotides, and reagents

Wildtype (x^+^) T7 DNA pol was reconstituted by combining the catalytic subunit (T7 gp5) with the *E. coli* thioredoxin accessory protein (47, 48) and purified as previously described (10). Active pol was reconstituted using at least a twofold molar excess of thioredoxin relative to the pol. Bovine serum albumin, T4 polynucleotide kinase (T4 PNK), and dNTPs were purchased from New England Biolabs. Polyethyleneimine-cellulose TLC plates and dCMP were purchased from Sigma–Aldrich. Before using TLC plates, plates were developed with distilled water until the water reached the top of the plate. The plates were dried, the contaminants at the top were cut off, and the plates were stored at 4 °C until use. Oligonucleotides not containing tC^o^ were purchased from Integrated DNA Technologies with standard desalting and then further purified by denaturing PAGE in house to achieve greater than 98% full-length oligonucleotide, as determined by capillary electrophoresis. Oligonucleotides containing tC^o^ were purchased from Bio-Synthesis Corporation (www.biosyn.com) with PAGE purification. Phosphorothioate oligonucleotides were purchased as a racemic mixture of the two diastereomers and enzymatically processed to leave only the nonhydrolyzable S_p_ isomer (described later). All PAGE reagents and buffer components were purchased from either Fisher Scientific or Sigma–Aldrich.

### Enzymatic synthesis of hydrolysis-resistant phosphorothioate oligos

Hydrolysis-resistant oligonucleotides containing an S_p_ phosphorothioate linkage were prepared by mixing 0.5 μM T7 DNA pol, 0.1 mg/ml bovine serum albumin, 25 μM phosphorothioate oligo, and 12.5 mM Mg^2+^ in T7 reaction buffer to start the reaction, and the reaction was incubated at 20 °C for 45 min. The reaction was then quenched by adding EDTA to a final concentration of 0.25 M. Processing of the reactions followed different protocols for oligonucleotides containing tC^o^ and oligonucleotides without tC^o^.

#### Purification of enzymatic degradation reactions for oligos containing tC^o^

Proteins were inactivated and removed by incubating the quenched sample at 75 °C for 20 min and then filtering the sample through a 0.2 μm Whatman syringe filter. Buffer A-1 (25 mM Tris–HCl, pH 8.9) was added at a ratio of 2:1 Buffer A-1 to sample. Samples were loaded onto a Bio-Rad Uno Q-1 column connected to an AKTA Pure FPLC system (GE Healthcare), equilibrated in 10% Buffer B-1 (Buffer A-1 with 1 M NaCl). Absorbance was monitored at 260 nm. The column was washed with 10 ml of 30% Buffer B-1, and then bound oligos were eluted with a gradient from 30 to 60% Buffer B-1 over 40 ml, collecting 1 ml fractions. Fractions containing the full-length nonhydrolyzable oligo were buffer exchanged into distilled water by ultrafiltration with GE Healthcare Vivaspin 20 centrifugal concentrators (5 kDa molecular weight cutoff). The concentration of the purified and buffer exchanged oligonucleotides was determined by absorbance at 260 nm using the extinction coefficients given in Table 1 and stored at −20 °C.

#### Purification of enzymatic degradation reactions for oligos without tC^o^

Proteins were inactivated by incubation at 85 °C for 10 min, and the precipitated protein was removed by filtration through a 0.22 μM syringe filter (Whatman). The sample was diluted 20-fold with Buffer A-2 (10 mM NaOH, pH 12) and loaded onto an Uno Q-1 anion exchange column (Bio-Rad) equilibrated in Buffer A-2. The oligo was eluted with a gradient of 0 to 90% Buffer B-2 (Buffer A-2 with 1 M NaCl) over 40 ml. Fractions containing the purified hydrolysis-resistant oligonucleotide were neutralized and then extensively buffer exchanged into TE buffer (Tris and EDTA) on Vivaspin 20 centrifugal concentrators (GE Healthcare) before determining the concentration using the extinction coefficients at 260 nm in Table 1 and storage at −20 °C. MALDI-MS was used to verify full-length phosphorothioate DNA obtained after the enzymatic degradation reaction using previously established methods (11).

### ^32^P labeling of tC^o^ oligonucleotide

Oligonucleotides containing tC^o^ were labeled with ^32^P for rapid-quench assays with T4 PNK by mixing 40 μM oligo, [γ-^32^P]-ATP, and T4 PNK (25 units) in T4 reaction buffer (supplied by NEB) in a volume of 40 μl at 37 °C for 1 h. T4 PNK was inactivated by incubation at 65 °C for 20 min. One microliter of the reaction was spotted on a polyethyleneimine-cellulose TLC plate, and excess ATP was removed from the sample with Oligo Clean and Concentrator spin columns (Zymo). One microliter of the cleaned-up reaction was spotted on a separate lane of the TLC plate and dried. The TLC plate was developed in 375 mM phosphate buffer, pH 4.0, dried, and then visualized with phosphorimaging. The concentration of labeled oligo was determined with the formula C_1_V_1_ = C_2_V_2_, and the purified oligo was stored at −20 °C until annealing with the template strand (described previously) for use in kinetics assays.

### Kinetic experiments

Kinetic experiments were performed in T7 reaction buffer (40 mM Tris–HCl [pH 7.5], 50 mM NaCl, 1 mM EDTA, and 1 mM DTT) (8, 9) at 20 °C. All reactions contained a final concentration of 12.5 mM Mg^2+^; however, some experiments had Mg^2+^ in both syringes, whereas other experiments had the enzyme and DNA in one syringe and Mg^2+^ was mixed in from the other syringe. In either case, the final Mg^2+^ concentration during the reaction was 12.5 mM. Concentrations of reaction components given in the text are final concentrations after mixing unless otherwise noted.

#### Rapid-quench experiments

Rapid-quench experiments were performed with T7 reaction buffer without Mg^2+^ in the drive syringes and 0.6 M EDTA in the quench syringe, with the circulating water bath set to 20 °C. Formamide loading buffer (5% [w/v] sucrose, 90% [v/v] formamide, 10 mM EDTA, 0.025% [w/v] bromophenol blue, and 0.025% [w/v] xylene cyanol) was added to each sample at a 1:2 ratio of loading buffer to sample. Samples were heated at 98 °C for 5 min and separated on 15% polyacrylamide sequencing gels containing 7 M urea for approximately 3 h at 50 °C. For samples with ^32^P-labeled oligos, gels were dried and then visualized by phosphorimaging after overnight exposure. For samples with FAM-labeled DNA, gels were scanned on a Typhoon FLA 9500 laser scanner (GE Healthcare) with the FAM fluorescence filter. For both ^32^P experiments and FAM experiments, gels were quantified with ImageQuant software (GE Healthcare). All rapid-quench experiments with FAM-labeled DNA were performed on at least two separate occasions to ensure reproducibility. Rapid-quench experiments with ^32^P-labeled tC^o^ oligos were reproduced with FAM-labeled substrates on at least two separate occasions with similar results.

#### Stopped-flow experiments

Stopped-flow experiments were performed on an SF-300× instrument (KinTek Corp) with a dead time of 1.3 ms using a 150-W xenon lamp (Hamamatsu) as the light source. All stopped-flow traces in the main text are an average of at least eight individual traces, and each experiment was repeated on at least three separate occasions to ensure reproducibility. Fluorescence anisotropy experiments with FAM-labeled oligos were performed with the anisotropy kit for the SF-300× instrument, including vertical and horizontal emission polarizers and a Glan-Taylor excitation polarizer. Polarizers were calibrated with a solution of glycogen in water using previously established methods (49). Before data collection, the G-factor was measured and applied to subsequent traces. Anisotropy experiments were performed with an excitation wavelength of 470 nm with a slit width of 3.16 mm, and emission was monitored with 520 nm filters with a 15 nm bandpass. Fluorescence experiments with tC^o^-labeled oligonucleotides were performed with excitation at 295 nm with a slit width of 1.56 mm. Emission was monitored at 445 nm with a 45 nm bandpass. All fluorescence experiments were performed on at least two separation occasions to ensure reproducibility.

Equilibrium titrations were performed with the TMX titration module for the SF-300× instrument. Titrations were performed with 280 μl of a solution of tC^o^ DNA in a cuvette in a temperature-controlled cuvette chamber, and 20.5 μl of titrant (enzyme) was added from a Hamilton syringe over the course of 5 min. Excitation was at 295 nm with a slit width of 1.56 mm. Emission was monitored with a 445 nm filter with a 45 nm bandpass. The reaction was mixed continuously with a micro stir bar in the cuvette, and the titration data were corrected for the small dilution before further manipulation. Because the sample was excited at 295 nm and the enzyme (FRET donor) was the titrant, the raw data showed the inverse of an inner filter effect, with increasing fluorescence with added enzyme because of the tryptophan residues acting as the FRET pair donor. To correct for the fluorescence artifact, first the data corrected for the small dilution were fit in KinTek Explorer using the following observable, where *a* is the overall scaling factor, *b* is the scaling factor for the increase in fluorescence upon binding DNA, and ԑ is the extinction coefficient of the titrant.

$$observable=a*\left(D+\left(b*ED\right)\right)*\frac{1-10^{\varepsilon *\left(E+ED\right)}}{\ln\left(10\right)*-\varepsilon *\left(E+ED\right)}$$

The fit from KinTek Explorer gave a value for e of 0.805, which was then used to correct the raw data using the following equation:

CorrectedFluorescence=RawFluorescence1−10ε∗[T7DNAPAdded]ln(10)∗−ε∗[T7DNAPAdded]

The corrected data were then fit in KinTek Explorer using the observable:

Observable=a∗(D+(b∗ED))

The corrected data are shown in the main text.

### Software and figures

Data analysis was performed with the simulation software KinTek Explorer, version 10 (21, 22). Kinetics figures were prepared with KinTek Explorer software. Model scheme images were prepared with Inkscape software (https://www.inkscape.org). Structure figures were prepared with PyMOL (50).

### Data analysis in KinTek Explorer

Mechanism files with global fits and confidence contours derived in fitting the data for the three substrates described in this article are provided, as described in the supporting information. These files can be opened without needing a license using KinTek Explorer software available at https://kintekexplorer.com/downloads/. Models used for data fitting are given in the figures for the corresponding substrates. For phosphorothioate substrates, parallel models without hydrolysis steps were used with rate constants linked to rate constants for the kinetic schemes for the hydrolyzable substrates. Flux calculations described in the main text were performed using a dynamic partial derivative analysis during data fitting based on numerical integration of the rate equations using KinTek Explorer software (19, 21).

For conventional fitting of data using equations described in the figure legends, the analytical fit (*aFit*) function of KinTek Explorer was used. The equation QUOTE $y=A_{0}+A_{1}\left(1-e^{-b_{1}t}\right)$ was used for fits to a single exponential function, where *A*_0_ is the *y*-intercept, *A*_1_ is the amplitude, and *b*_1_ is the observed rate. The equation QUOTE $y=A_{0}+A_{1}\left(1-e^{-b_{1}t}\right)+A_{2}\left(1-e^{-b_{2}t}\right)$ was used for data fitting two exponentials where *A*_0_ is the *y*-intercept, *A*_1_ and *A*_2_ are the amplitudes, and *b*_1_ and *b*_2_ are the observed rates of the first and second phases, respectively. For data fitting three exponentials, the following equation was used: $y=A_{0}+A_{1}\left(1-e^{-b_{1}t}\right)+A_{2}\left(1-e^{-b_{2}t}\right)+A_{3}\left(1-e^{-b_{3}t}\right)$, where *A*_0_ is the *y*-intercept, *A*_n_ is the amplitude, and *b*_n_ is the observed rate of the nth exponential. The quadratic equation used was QUOTE $=A_{0}+A\frac{K_{d}+E+S-\sqrt{{\left(K_{d}+E+S\right)}^{2}+4ES}}{2E}$, where *A*_0_ is the *y*-intercept, *A* is the amplitude, *K*_*d*_ is the equilibrium dissociation constant, *E* is the enzyme concentration, and *S* is the substrate concentration. The equation used for the hyperbolic fit was QUOTE $y=A_{0}+\frac{A_{1}S}{K_{d}+S}$, where *A*_0_ is the *y*-intercept, *A*_1_ is the amplitude, *S* is the substrate concentration, and *K*_*d*_ is the equilibrium dissociation constant. The results from equation-based fitting ar e given in the figure legends but were used only to guide the development of the complete models derived by global data fitting. All lines given in Figure 2, Figure 4, Figure 6 were calculated by numerical integration from the global fits using the complete models.

## Data availability

All data are available in the supporting information in the form of KinTek Explorer mechanism files. These files can be opened (without needing a license) using KinTek Explorer software available at https://kintekexplorer.com, where video tutorials are also available to illustrate use of the software. The mechanism files also show how the data were modeled, and users can explore alternative models (19, 22).

## Supporting information

This article contains supporting information (9, 18, 19, 22).

## Conflict of interest

K. A. J. is the president of KinTek Corporation, which provided the RQF-3 rapid-quench flow and SF 300-X stopped-flow instruments and KinTek Explorer software used in this study. All other authors declare that they have no conflicts of interest with the contents of this article.
