# Supplementary material for: Kinetics of DNA strand transfer between polymerase and proofreading exonuclease active sites regulates error correction during high-fidelity replication
Source: J Biol Chem. 2022 Nov 24;299(1):102744. doi: 10.1016/j.jbc.2022.102744 (PMC9800556; doi:10.1016/j.jbc.2022.102744)
Supplement: Supplemental information [file mmc1.docx]

Supplemental information for:

**Kinetics of DNA strand transfer between polymerase and proofreading exonuclease active sites regulates error correction during high-fidelity replication**

**Tyler L. Dangerfield and Kenneth A. Johnson***

Department of Molecular Biosciences,

University of Texas, 2500 Speedway, Austin, TX 78712, USA


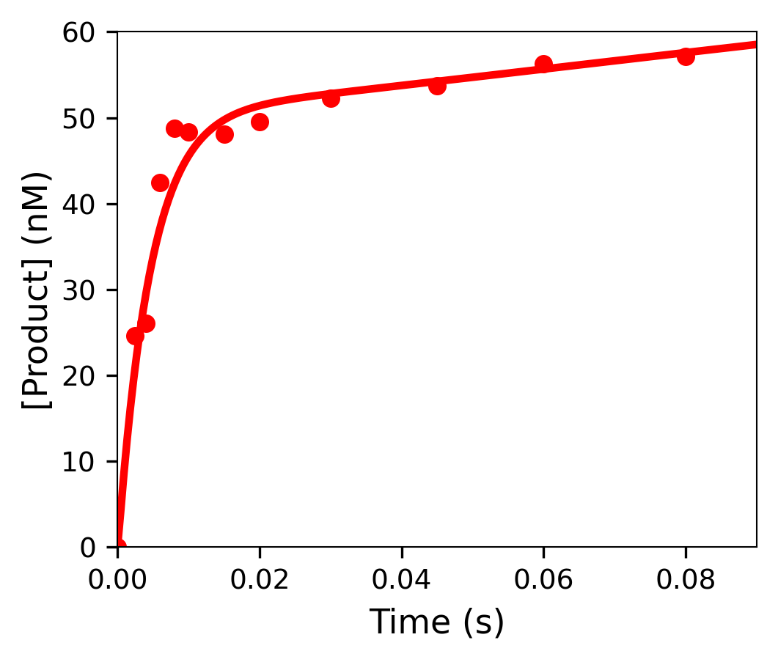


**Figure S1: Rapid quench pre-steady state burst control experiment on ^32^P-tC^o^ DNA.** A solution of 100 nM ^32^P-tC^o^ DNA (^32^P-tC^o^-27/45-P/T), 60 nM exo^-^ T7 DNA polymerase, 1.2 μM thioredoxin and 0.1 mg/ml BSA was mixed with 12.5 mM Mg^2+^ and 150 μM dATP to start the reaction. The reaction was performed at 4°C as described previously ([1](#_ENREF_1)) and time points were quenched with EDTA. Samples were separated by denaturing PAGE, the gel was dried, then visualized by phosphorimaging. The solid line through the data is the best fit by simulation in KinTek Explorer to a simple one step nucleotide binding and chemistry model.


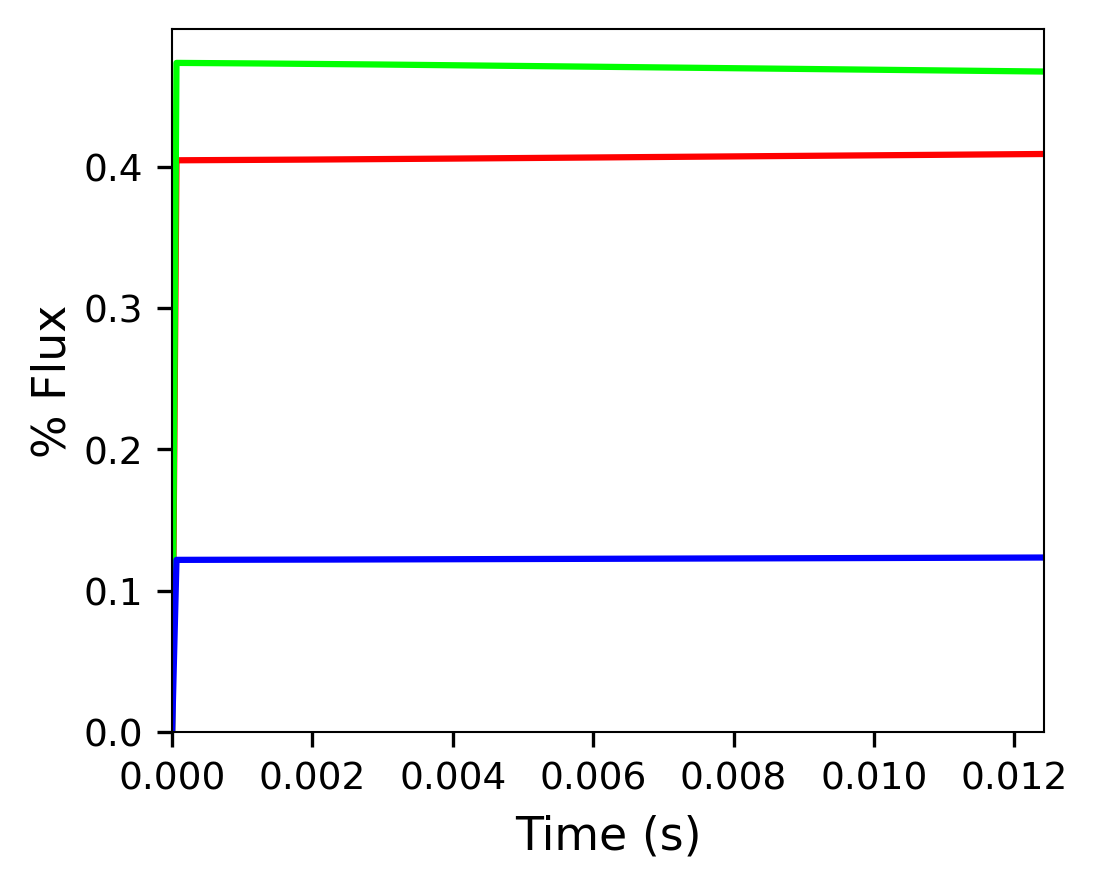


**Figure S2: Flux calculation for 3’ mismatch binding and excision experiment.** The fraction of the DNA that binds at the polymerase active site, intermediate site, and exonuclease active site are shown in red, green, and blue, respectively.


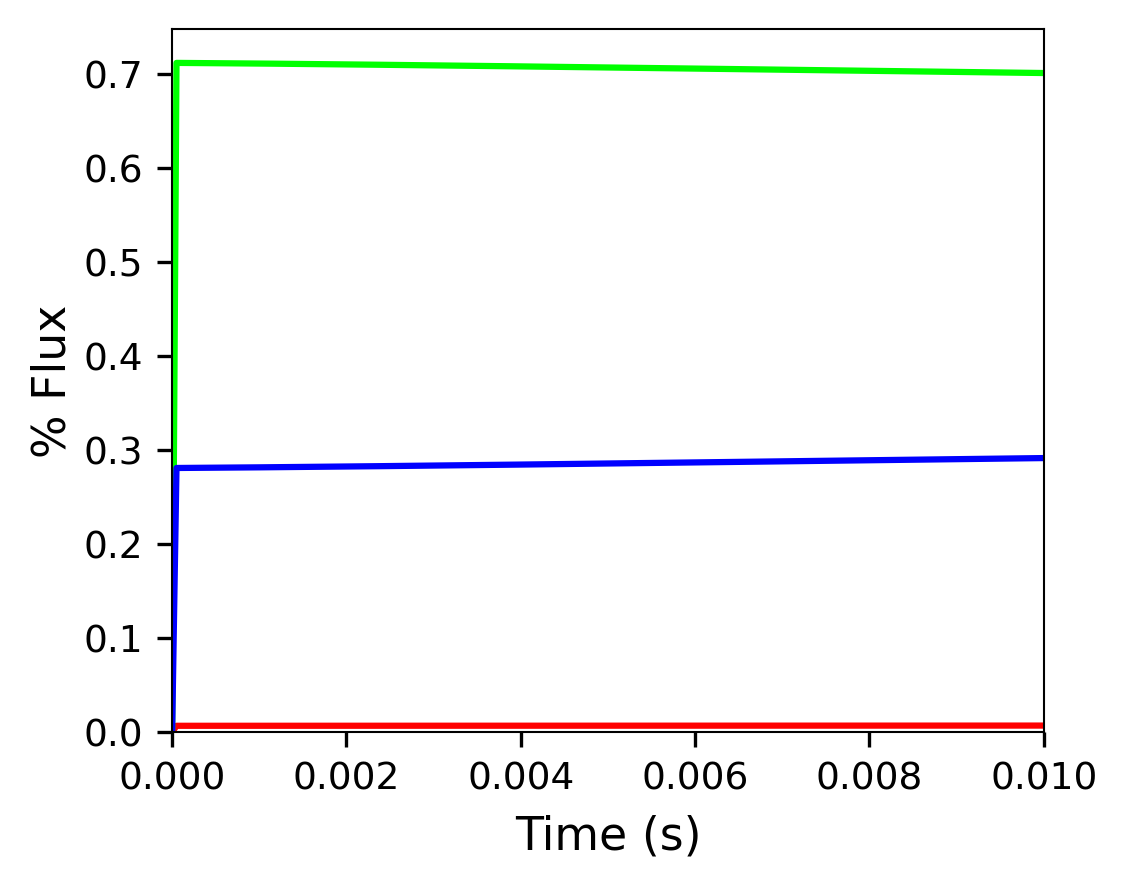


**Figure S3: Flux calculation for buried mismatch binding and excision experiment.** The fraction of the DNA that binds at the polymerase active site, intermediate site, and exonuclease active site are shown in red, green, and blue, respectively.

Supplemental KinTek Explorer Mechanism Files

These files can be opened using KinTek Explorer software ([2-4](#_ENREF_2)) available for both Windows and Mac OSX at https://kintekexplorer.com. A software license is not required to open any existing mechanism files. Instructions are included in the /documents directory with the software and tutorials are available online.

**jbc_00102744_mmc2.mec**

Contains the mechanism file for the ssDNA data in Figure 2 and the corresponding FitSpace calculation in Figure 3.

**jbc_00102744_mmc3.mec**

Contains the mechanism file for the 3’ mismatch data in Figure 4 and the corresponding FitSpace calculation in Figure 5. Flux calculations described in the text and shown in Figure S2 are also given in the mechanism file.

**jbc_00102744_mmc4.mec**

Contains the mechanism file for the buried mismatch data in Figure 6 and the corresponding FitSpace calculation in Figure 7. Flux calculations described in the text and shown in Figure S3 are also given in the mechanism file.

Reference Cited

1. Dangerfield, T. L., and Johnson, K. A. (2021) Conformational dynamics during high-fidelity DNA replication and translocation defined using a DNA polymerase with a fluorescent artificial amino acid. *J. Biol. Chem.* **296**, 100143

2. Johnson, K. A. (2019) *Kinetic Analysis for the New Enzymology*, KinTek Corporation, Austin

3. Johnson, K. A., Simpson, Z. B., and Blom, T. (2009) FitSpace Explorer: An algorithm to evaluate multidimensional parameter space in fitting kinetic data. *Anal. Biochem.* **387**, 30-41

4. Johnson, K. A., Simpson, Z. B., and Blom, T. (2009) Global Kinetic Explorer: A new computer program for dynamic simulation and fitting of kinetic data. *Anal. Biochem.* **387**, 20-29
